# ASAOT: an accentuated structure-differentiated asymmetric optimal transport model for cell type and single-cell spatial mapping

**DOI:** 10.1093/bib/bbag615

**Published:** 2026-11-13

**Authors:** Ziyan Sun, Zhixin Shi, Yuan Zhang, Mengdi Nan, Jie Gao

**Affiliations:** School of Mathematics and Data Science, Jiangnan University, 1800 Lihu Avenue, Binhu District, Wuxi, Jiangsu 214122, China; School of Mathematics and Data Science, Jiangnan University, 1800 Lihu Avenue, Binhu District, Wuxi, Jiangsu 214122, China; School of Mathematics and Data Science, Jiangnan University, 1800 Lihu Avenue, Binhu District, Wuxi, Jiangsu 214122, China; School of Mathematics and Data Science, Jiangnan University, 1800 Lihu Avenue, Binhu District, Wuxi, Jiangsu 214122, China; School of Mathematics and Data Science, Jiangnan University, 1800 Lihu Avenue, Binhu District, Wuxi, Jiangsu 214122, China

**Keywords:** spatial transcriptomics, spatial deconvolution, optimal transport, cell-type proportion, single-cell spatial mapping

## Abstract

Spatial transcriptomics (STs) characterizes spatiotemporal gene expression and facilitates analysis of cellular architecture and cell–cell crosstalk within tissue microenvironments. However, existing spatial deconvolution methods employ globally fixed smoothing without local sharpening, which blurs cellular boundaries and impedes reconstruction of layered, heterogeneous cellular patterns in brain tissue. Here, we present Accentuated Structure-differentiated Asymmetric Optimal Transport (ASAOT), a deconvolution framework built on structure-enhanced asymmetric optimal transport. Coupled with spatial neighborhood constraints, ASAOT introduces a differentiated accentuated sharpening term to maintain global spatial continuity, enhance boundary detection, and preserve spot-level expression heterogeneity. Using asymmetric optimal transport, the framework jointly estimates spot-wise cell-type proportions and performs spatial assignment of reference single-cell coordinates. Benchmarks on diverse simulated datasets demonstrate that ASAOT achieves competitive overall accuracy and robustness across moderate spatial resolutions, with the best average comprehensive rank among tested methods. Applications to mouse olfactory bulb and cerebral cortex datasets demonstrate that ASAOT reduces signal diffusion and boundary distortion, effectively recovering layered brain anatomy and stratified cell distributions to build interpretable spatial maps of reference single-cell profiles. Collectively, ASAOT addresses critical drawbacks of conventional methods via a sharpening regularization term, serving as a reliable tool for STs analysis and mechanism exploration of tissue spatial organization.

## Introduction

Tissues and organs consist of multiple cell types, which exhibit substantial differences in size, morphology, transcriptional signatures, and *in situ* spatial distribution. Continuous intercellular signal communication collectively governs the overall biological functions of tissues [1]. Single-cell RNA-sequencing (scRNA-seq) enables accurate identification of all cell subtypes and characterization of single-cell transcriptional heterogeneity, yet loses the native spatial arrangement of cells during tissue dissociation [2]. Spatial transcriptomics (STs) technologies can preserve the intact *in situ* spatial coordinates of tissues, but are generally constrained by a trade-off between spatial resolution and detected gene throughput [3].

Current ST technologies are broadly categorized into two classes: high-throughput, low-resolution spatial capture sequencing techniques and high-resolution, low-throughput *in situ* imaging approaches [4]. Sequencing-based platforms, including 10× Genomics Visium [5] and Slide-seq [6], achieve transcriptome-wide profiling via spatial barcoding. However, individual spatial spots typically capture mixed signals originating from multiple cells, failing to attain single-cell resolution. Imaging-based spatial methods such as multiplexed error‑robust fluorescence in situ hybridization (MERFISH) [7] enable subcellular localization but suffer from a limited number of detectable genes. Accordingly, integrating scRNA-seq data with transcriptome-wide STs datasets represents an indispensable strategy for dissecting tissue spatial microenvironments, cellular neighborhoods, and hierarchical tissue heterogeneity [8].

Existing analytical frameworks integrating single-cell and STs can be divided into cell mapping methods and spatial deconvolution approaches [9]. Methods such as novoSpaRc (an optimal-transport-based method for spatial reconstruction of single-cell transcriptomes) [10], Tangram (a deep learning-based spatial mapping and cell-type deconvolution method) [11], CellTrek [12], and CytoSPACE (cellular Spatial Positioning Analysis via Constrained Expression alignment) [13] fall into the cell mapping category, which aims to infer the *in situ* spatial coordinates of single cells. Among them, novoSpaRc [10] and Tangram [11] directly output spatial positions of single cells yet exhibit low algorithmic fault tolerance, minor sequencing noise can readily trigger widespread biases in cell localization. CellTrek [12] establishes direct correspondence between single cells and spatial spots, whose localization accuracy heavily relies on the cellular purity of spatial spots and yields considerable errors in mixed regions. CytoSPACE [13] requires precomputed deconvolution results as input, which makes it prone to inheriting upstream decomposition errors and further impairs the accuracy and robustness of spatial localization.

Spatial deconvolution treats the gene expression of each spot as a weighted average of transcriptional signals originating from diverse cell types, and decomposes cell-type fractions within individual spots using single-cell reference profiles [14]. Stereoscope [15], Robust cell type decomposition (RCTD) [16], Cell2location [17], and SPOTlight [18] employ probabilistic modeling to estimate cell-type proportions. Redeconve [19] is specially designed to mitigate gene collinearity issues in high-resolution datasets. Probabilistic-based deconvolution with spot-adaptive cell type signatures (PANDA) [20] and Deconvolution of spatial transcriptomics profiles using variational inference (DestVI) [21] simultaneously enable cell proportion prediction and characterization of subtype-specific transcriptional heterogeneity. Unified single‑cell data integration framework (UniPort) [22] leverages optimal transport for cross-modal integration and spatial deconvolution, however, its data alignment solely relies on transcriptional similarity without incorporating tissue spatial topology priors. Spatial transcriptomics deconvolution by topic modeling (STRIDE) [23] estimates cell proportions via topic modeling and subsequently performs spatial domain segmentation using coordinate information, where spatial priors are not involved in regulating cell proportion estimation. To further improve spatial resolution accuracy, Conditional autoregressive-based deconvolution (CARD) [24] integrates a spatial neighborhood regularizer within a Bayesian framework, where the similarity of cellular compositions between adjacent spots is constrained to achieve spatially informed smoothing. Spatially weighted poisson-gamma regression (SONAR) [25] optimizes estimations by exploiting information from neighboring spots and adopts elastic weighting to filter dissimilar neighbors. Nevertheless, its bandwidth parameter that defines neighborhood range remains globally fixed across the entire tissue section. Spatially weighted optimal transport (SWOT) [26] embeds spatial continuity constraints into an optimal transport framework to enforce spatial consistency of cellular transport distributions among neighboring spatial spots. Although all the above methods improve deconvolution accuracy by harnessing spatial information, most adopt globally uniform spatial constraint parameters. This tends to oversmooth subtle compositional differences at tissue boundaries and cell-type transition zones, resulting in diffused boundary signals. Consequently, they struggle to simultaneously satisfy continuity within layered tissue regions and specificity at tissue boundaries. Furthermore, the vast majority of deconvolution tools can only output cell-type proportions within each spatial spot. They fail to estimate cell-level abundance, making it difficult to generate probabilistic single-cell spatial assignments for downstream spatial neighborhood analysis [12]. This limitation restricts their potential to explore cellular neighborhoods, motivating the development of methods that can both deconvolve and spatially map single cells.

In this study, we develop Accentuated Structure-differentiated Asymmetric Optimal Transport (ASAOT), a framework that uses single-cell transcriptomes as a reference and integrates spatial structural information to perform spot-wise cell-type deconvolution, estimate cellular abundance per spot, and infer spatial coordinates for reference single-cell profiles. ASAOT possesses two core innovations. First, instead of adopting a globally uniform smoothing scheme, we introduce a differentiated sharpening regularization term within the optimal transport framework to enhance discriminative patterns of spatially distributed cell types, effectively mitigating boundary blurring and signal diffusion caused by over-smoothing. Second, we perform resolution-dependent estimation of the total cell count per spot. Combined with spatial neighborhood information, this step recovers biologically plausible single-cell coordinates and maps reference single-cell profiles onto spatial positions from mixed spot-level signals, effectively mitigating the resolution limitation of conventional STs. Systematic comparisons across multiple simulated datasets demonstrate that ASAOT achieves competitive overall accuracy and robustness across moderate spatial resolutions, with the best average comprehensive rank among the eight tested deconvolution algorithms. When applied to real ST datasets of the mouse olfactory bulb (MOB) and cerebral cortex, ASAOT effectively recapitulates the layered anatomical architecture of brain tissue and quantifies the depth-wise distribution and colocalization features of neurons, glial cells, and vascular cells. Furthermore, single-cell coordinate inference facilitates characterization of spatial neighboring compositions of cortical inhibitory neurons.

## Methods

### Overview of the ASAOT model

ASAOT is a STs deconvolution model that uses scRNA-seq data as a reference. It integrates gene expression profiles and spatial coordinate information from ST datasets to infer cell-type composition and generate probabilistic spatial assignments for reference single-cell profiles. By introducing a differentiated sharpening regularization term, ASAOT accentuates spatial distribution disparities among distinct cell types, alleviates boundary blurring, and improves the accuracy of cell-type proportion deconvolution. The model ultimately outputs cell-type fractions for each spatial spot as well as an inferred spatial map of reference single-cell profiles.

ASAOT consists of two core modules: the optimal transport module and the cell mapping module. The first module obtains a probabilistic matching transport plan between single cells and spatial spots by solving an unbalanced structured optimal transport problem that incorporates a sharpening term. On the basis of the transport plan and cell-type labels, the second module sequentially estimates cell-type proportions, resolution-dependent cellular abundance per spot, and generates spatial coordinates for each reference single-cell profile.

### Input datasets and mathematical notation

First, let $\mathbf{\mathcal{G}}=\left\{1,2,\kern-1pt..,G\right\}$ denote the set of all genes, $\mathbf{\mathcal{C}}=\left\{1,2,\kern-1pt..,C\right\}$ the set of all single cells, $\mathbf{\mathcal{K}}=\left\{1,2,..,K\right\}$ the set of all cell types,${\mathbf{\mathcal{C}}}_{\boldsymbol{k}}=\left\{1,2,\kern-1pt..., {C}_k\right\}$ the set of all cells belonging to cell type *k* and$\mathbf{\mathcal{I}}=\left\{1,2,\kern-1pt..,I\right\}$ the set of all spatial spots. We then input the single-cell transcriptomic expression matrix $\boldsymbol{S}$, where ${S}_{gc}^{(k)}$ represents the expression count of gene $g\in \mathbf{\mathcal{G}}$ in single cell $c\in{\mathbf{\mathcal{C}}}_k$ assigned to cell type $k\in \mathbf{\mathcal{K}}$. The ST expression matrix is denoted as $\boldsymbol{T}$, where ${T}_{gi}$ corresponds to the expression count of gene *g* at spatial spot $i\in \mathbf{\mathcal{I}}$. The coordinate vector $\left\{\left({x}_i,{y}_i\right),i\in \mathbf{\mathcal{I}}\right\}$ defines the spatial location of spatial spot *i* on the tissue section.

Using the input information, we construct three normalized distance matrices to align single-cell expression profiles with spot expression profiles. Joint structural constraints derived from the expression space and physical space provide prior information for the optimal transport plan governing cell-to-spot mapping.

### Construction of three distance-matrix components

First, to preserve the expression neighborhood relationships among single cells during transport—under the assumption that cells with similar expression tend to map to spatially adjacent spots [27], we build a K-nearest-neighbor undirected graph based on single-cell expression vectors. The Dijkstra algorithm is adopted to compute the shortest path over the graph, yielding the intercellular distance matrix ${\boldsymbol{M}}_{\boldsymbol{se}}$, which quantifies the gene expression similarity between any pair of single cells.

Next, cells are more likely to be assigned to spatial spots whose expression signatures most closely match their own. Accordingly, we compute the cell-to-spot distance matrix based on dissimilarity metrics derived from gene expression profiles [1-Pearson correlation coefficient (PCC) as the default; cosine distance or Euclidean distance is also available], which serves as the transport cost matrix $\boldsymbol{M}$ for assigning cells to spatial spots in the subsequent optimal transport formulation.

Finally, relying solely on physical coordinates fails to distinguish differences in cell-type composition across distinct tissue regions [28]. Instead of directly adopting raw coordinate distances, we integrate gene expression-based pre-clustering of spatial spots to construct an enhanced spatial distance matrix ${\boldsymbol{M}}_{\boldsymbol{cost}}$. Specifically, we first perform pre-clustering on the gene expression profiles of spatial spots using the Leiden algorithm to obtain cluster labels. By default, the clustering resolution is tuned such that the total number of clusters equals the total number of reference cell types. As an optional alternative, the pre-cluster number may be selected independently from the ST expression profiles via the elbow method or silhouette coefficient in a reference-free preprocessing step (see Robustness analysis of pre-clustering resolution). For all pairs of spots $\left(i,j\right)$, we categorize them into four scenarios according to whether their coordinate distance $p{s}_{ij}$ lies within the bandwidth *b* and whether the two spots belong to the same pre-cluster. Different distance calculation strategies are assigned to each scenario (see Supplementary Note S1). This approach more accurately characterizes the genuine biological associations between spatial spots within heterogeneous tissues and acts as a structural prior incorporated into the subsequent optimal transport framework [26].

### Objective function of ASAOT

Upon obtaining the three aforementioned distance matrices, ASAOT solves the following unbalanced structured optimal transport problem equipped with a differentiated sharpening regularization term to derive the cell-to-spot transport plan ${\boldsymbol{P}}^{\ast }$: 


(1) \begin{align*}{\boldsymbol{P}}^{\ast }=&\,\underset{P\ge 0}{\mathit{\arg}\min}\left(1-\alpha \right)\left\langle \boldsymbol{M},\boldsymbol{P}\right\rangle +\lambda \left[{D}_{KL}\left(\boldsymbol{P}{\mathbf{1}}_I,\boldsymbol{a}\right)+{D}_{KL}\left({\boldsymbol{P}}^T{\mathbf{1}}_C,\boldsymbol{b}\right)\right] \nonumber \\ &+\, \alpha \cdotp GW\left(\boldsymbol{P};{\boldsymbol{M}}_{se},{\boldsymbol{M}}_{\cos t}\right)+\beta \cdotp{R}_{sharp}\left(\boldsymbol{P};{\boldsymbol{M}}_{se},{\boldsymbol{M}}_{\cos t}\right)+\varepsilon H\left(\boldsymbol{P}\right), \end{align*}

where $\left\langle \cdotp, \cdotp \right\rangle$ denotes the Frobenius inner product, and $\boldsymbol{P}\in{\mathbb{R}}_{+}^{C\times I}$ represents the transport plan, with each entry ${P}_{ci}$ corresponding to the probability of assigning cell *c* to spatial spot *i*. $\boldsymbol{a}=\frac{1}{C}{\mathbf{1}}_C$ and $\boldsymbol{b}=\frac{1}{I}{\mathbf{1}}_I$ are the initial mass distributions of cells and spatial spots, respectively. $\lambda, \alpha, \beta, \varepsilon$ correspond to the unbalanced penalty coefficient, structural term weight, sharpening term weight, and entropy regularization coefficient. ${D}_{KL}$ denotes the Kullback–Leibler (KL) divergence, which relaxes the mass conservation constraint. The structural term adopts the Gromov–Wasserstein distance [29] to maintain consistency between the intrinsic relationships among single cells and those among spatial spots. $GW=\sum_{c,{c}^{\prime }}\sum_{i,{i}^{\prime }}L\left({\boldsymbol{M}}_{se}\left(c,{c}^{\prime}\right),{\boldsymbol{M}}_{\cos t}\left(i,{i}^{\prime}\right)\right){P}_{ci}{P}_{c^{\prime }{i}^{\prime }}$, where ${\boldsymbol{M}}_{se}\left(c,{c}^{\prime}\right)$ measures the distance between cell *c* and ${c}^{\prime }$ in the single-cell expression space, and ${\boldsymbol{M}}_{\cos t}\left(i,{i}^{\prime}\right)$ denotes the distance between spatial spot *i* and spot ${i}^{\prime }$ in the enhanced spatial space. The loss function is denoted as $L\left(x,y\right)=\frac{1}{2}{\left(x-y\right)}^2$ . To sharpen tissue boundaries and mitigate spatial blurring between distinct cell-type domains, we introduce an additional regularization term ${R}_{sharp}=\sum_{c,{c}^{\prime }}\sum_{i,{i}^{\prime }}L\left({\boldsymbol{M}}_{se}\left(c,{c}^{\prime}\right),{\boldsymbol{M}}_{\cos t}\left(i,{i}^{\prime}\right)\right){P}_{ci}{P}_{c^{\prime }{i}^{\prime }}$ that shares the pairwise summation structure of the GW term but uses the original (unscaled) spatial distance matrix and a distinct gradient formulation. The gradient of ${R}_{sharp}$ with respect to the transport plan $\boldsymbol{P}$ is then: $\nabla{R}_{sharp}=2\beta \left({\boldsymbol{C}}_0-{\boldsymbol{M}}_{se}\boldsymbol{P}{\boldsymbol{M}}_{\cos t}^T\right)$, where ${\boldsymbol{C}}_0={\boldsymbol{M}}_{se}^2\boldsymbol{a}{\mathbf{1}}_I^T+{\mathbf{1}}_C{\boldsymbol{b}}^T{\left({\boldsymbol{M}}_{\cos t}^2\right)}^T$ denotes the auxiliary matrix. In contrast, the *GW* term in our formulation is defined with the same loss function and the same distance matrices, but its gradient follows the standard *GW* form: $\nabla GW=2\alpha \left({\boldsymbol{C}}_0-2{\boldsymbol{M}}_{se}\boldsymbol{P}{\boldsymbol{M}}_{\cos t}^T\right)$, where the factor 2 in the second term arises from the standard derivation of the *GW* gradient with the squared loss. Functionally, the *GW* term is designed to preserve inter-relationships across single-cell and spot metric spaces through optimal transport mapping. It embeds intrinsic structural information from both spaces and maintains large-scale global spatial continuity for layered tissue architecture. In contrast, ${R}_{sharp}$ is a dedicated sharpening regularization term. Its core purpose is to amplify signal contrast at tissue boundaries and mitigate boundary blurring between dissimilar cell-type domains. These two terms cannot be simply merged into a single unified composite distance term. Combining them would entangle the global structure-matching objective and sharpening-driven boundary enhancement objective within one loss component. Consequently, we would lose the ability to independently tune their respective strengths via hyperparameters *α* and *β*. Maintaining two separate loss terms enables flexible trade-off between global spatial continuity and boundary sharpness, which is critical for accurate deconvolution of complex layered tissue structures. $H\left(\boldsymbol{P}\right)=\sum_{c,i}{P}_{ci}\log{P}_{ci}$ represents the entropy regularization term, which accelerates the convergence of Sinkhorn iterations.

### Optimization via conditional-gradient-Sinkhorn algorithm

Equation (1) is solved via the conditional gradient algorithm. Each iteration consists of three steps: Step 1: Compute the gradients of *GW* and ${R}_{sharp}$ with respect to $\boldsymbol{P}$; Step 2: Construct the composite cost matrix: ${\boldsymbol{M}}^{(t)}=\boldsymbol{M}+\alpha{\nabla}_{GW}+\beta{\nabla}_{R_{sharp}}$, and solve for $\overset{\sim }{\boldsymbol{P}}= Sinkhorn\left(\boldsymbol{a},\boldsymbol{b},{\boldsymbol{M}}^{(t)},\varepsilon, \lambda \right)$ using the Sinkhorn algorithm; Step 3: Perform line search to determine the optimal step size *η* and update ${\boldsymbol{P}}^{\left(t+1\right)}={\boldsymbol{P}}^{(t)}+\eta \left(\overset{\sim }{\boldsymbol{P}}-{\boldsymbol{P}}^{(t)}\right)$. The above procedure is repeated until convergence. By default, the maximum number of iterations is set to 1000 with a tolerance $1{0}^{-6}$, and the optimal transport plan ${\boldsymbol{P}}^{\ast }$ is output upon termination.

### Cell mapping module

Leveraging the probabilistic transport matrix ${\boldsymbol{P}}^{\ast }$ converged from the optimal transport module, together with pre-annotated cell-type labels of scRNA-seq single cells, we sequentially perform cell-type fraction estimation, cell count estimation per spot, and inference of spatial coordinates and expression profiles for reference single-cell profiles, ultimately producing a biologically plausible spatial-resolution map of reference single-cell profiles.

(1) Cell-type fraction estimation

Let $\left|{\mathbf{\mathcal{C}}}_{\boldsymbol{k}}\right|$ denote the total number of single cells within set ${\mathbf{\mathcal{C}}}_{\boldsymbol{k}}$. For any spatial spot $i\in \mathbf{\mathcal{I}}$, we sum the transport probabilities from all single cells of cell type *k* toward spot *i*. The raw fraction of cell type *k* at spot *i* is obtained by dividing this summed probability by the total cell count $\left|{\mathbf{\mathcal{C}}}_{\boldsymbol{k}}\right|$ of cell type *k*:


(2) \begin{equation*} {F}_{ik}=\frac{\sum_{c\in{\mathbf{\mathcal{C}}}_{\boldsymbol{k}}}{P}_{ci}^{\ast }}{\left|{\mathbf{\mathcal{C}}}_{\boldsymbol{k}}\right|}, \end{equation*}

To filter tiny numerical noise introduced by floating-point operations, we perform min-cut pruning with a fixed threshold: all fractions below the threshold are set to zero. Each spatial spot is then normalized row-wise to ensure that the sum of fractions of all cell types within an individual spot equals one, yielding the normalized cell-type fraction matrix $\boldsymbol{F}\in{\mathbb{R}}^{I\times K}$ as the final output.

(2) Estimation of cell number per spatial spot

In STs datasets, zero-inflated expression profiles are known to correlate negatively with local cellular density [30, 31]. Motivated by this observation, we posit that spatial spots exhibiting a larger fraction of zero-read counts are likely to harbor fewer cells, and conversely (see Supplementary Fig. S1).

We calculate ${z}_i$, the proportion of zero-expression counts across all genes for each spatial spot *i*. Let ${n}_i$ denote the total number of cells residing in spot *i*. The initial estimate of cellular abundance at each spot is derived via a log-linear model: ${n}_i=-10\ln \left({z}_i+0.001\right)-1$, with a constant of 0.001 added to avoid computational errors caused by zero input to the logarithmic function. The slope (−10) and intercept (−1) hyperparameters were tuned empirically using our simulated benchmark datasets, so that the resulting curve recapitulates the anticipated negative association between zero-expression fraction and spot-level cell density (see Supplementary Fig. S1). Before platform-aware bounds are enforced, this set of parameters reliably generates preliminary cell-count estimates that fall within biologically plausible limits across most zero-proportion values. Importantly, this log-linear function captures only a broad overall trend and cannot deliver precise cell-count predictions for every individual spot. Variability in messenger RNA (mRNA) capture efficiency and local transcriptional heterogeneity across spatial locations can break this approximate relationship. Since different spatial-omics platforms carry distinct physical spot dimensions, each technology has its own natural capacity for cell capture.

A biologically plausible range of cell numbers is defined according to the native resolution of different STs platforms: the range is constrained to $1\leqslant{n}_i\leqslant 10$ for the 10× Visium platform and $1\leqslant{n}_i\leqslant 3$ for the Slide-seq platform. Spots from 10× Visium, with a diameter of 55 μm, commonly contain between 1 and 10 cells [5], whereas ~10 μm-sized Slide-seq droplets usually hold 1–3 cells [32]. To guarantee biologically meaningful outputs, we clamp preliminary estimations within these technology-dependent cell-number boundaries. If the preliminary estimated value falls outside the predefined interval, it is replaced by a randomly sampled integer within the valid range to obtain the finalized cell count ${n}_i$ for spot *i*. This post-estimation adjustment reduces the algorithm’s reliance on the initial log-linear assumption and harmonizes inferred cell abundances with the well-characterized physical features of each STs platform. Estimation accuracy of this log-linear approach is resolution-dependent, achieving reliable predictions under moderate spot densities while exhibiting elevated bias for ultra-high-density datasets (see Supplementary Table S1).

(3) Assignment of reference single-cell spatial coordinates and expression profiles

Taking the original 2D coordinate $\left({x}_i,{y}_i\right)$ of spatial spot *i* as the center, we set half the distance from spot *i* to its nearest neighboring spot as the circular sampling radius. To correct the inherent radial central aggregation bias introduced by polar coordinate sampling, a square-root transformation is applied to radial distances, while angles are uniformly and randomly sampled within the interval $\left[0,2\pi \right]$. Two-dimensional spatial coordinates for ${n}_i$ virtual single cells are thereby generated. Single cells are sorted in descending order according to the transport probability ${P}_{ci}^{\ast }$, and the top ${n}_i$ cells with the highest probabilities are assigned to the circular region of spot *i*. The raw gene expression counts of the corresponding single cells from the scRNA-seq reference are directly assigned to these spatially mapped cells. After iterating over all spots, we finally obtain a spatial dataset with 2D spatial coordinates for mapped reference single-cell profiles.

### Hyperparameter selection

The selection criteria and tuning strategies for most hyperparameters of ASAOT (including the number of k-nearest neighbour (KNN) neighbors, distance metric, spatial neighborhood bandwidth *b*, ${\rho}_{indiff}$, ${\rho}_{outsam}$, unbalanced penalty coefficient *λ*, entropy regularization coefficient *ɛ*, maximum iteration number, convergence tolerance, and min-cut pruning threshold) can follow the configuration scheme of SWOT, a conventional structured optimal transport model [26]. In this section, we provide a detailed description exclusively for *β*, the newly introduced specific hyperparameter of ASAOT.

The hyperparameter *β* governs the weight of the sharpening term within the overall objective function and directly modulates the spatial aggregation degree of the transport plan. When *β =0*, ASAOT degenerates into a conventional optimal transport model. When *β >0*, the sharpening gradient takes effect and imposes aggregation-repulsion effects on the pre-clustering prior embedded in the inter-spot distance matrix. Based on systematic grid-search sensitivity analysis across simulated datasets, the optimal *β* is highly adaptive to the intrinsic signal characteristics originating from different ST technologies. For MERFISH-derived data, which frequently exhibit signal diffusion between adjacent spatial spots, we recommend a mild setting of *β =0.001*. Under this configuration, the sharpening term sharpens cell-type spatial boundaries effectively without impairing the convergence of the optimal-transport objective by over-penalization. For high-quality sequential fluorescence in situ hybridization (seqFISH-like) datasets with intrinsically crisp anatomical boundaries, the sharpening regularization brings marginal additional gains, and *β =0* is preferred. If *β* is set too small, the sharpening effect nearly vanishes, and boundary diffusion cannot be efficiently suppressed. Consequently, the model fails to achieve satisfactory boundary discrimination performance in layered tissues or heterogeneous regions with distinct boundaries. By contrast, an excessively large *β* allows the sharpening gradient to dominate optimization, which may lead to over-aggregation of the transport plan and further yield spatially discontinuous aberrant estimations.

### Robustness analysis of pre-clustering resolution

To address the potential risk that the spatial-cluster structural prior might be biased toward the scRNA-seq reference annotation, and to test whether our deconvolution conclusions remain valid under misspecified pre-cluster numbers, we performed a sensitivity-based robustness analysis on the MOB dataset [33]. The scRNA-seq reference of the MOB dataset contains five major annotated cell types, and this number serves as the baseline reference cell-type number in our analysis.

We systematically tested three representative Leiden clustering resolution values for the resolu_cluster parameter: 0.5, 0.9, and 1.2. These three settings correspond to three distinct pre-clustering scenarios: a lower clustering resolution yielding fewer spatial pre-clusters than the reference cell-type number (0.5), a moderate resolution producing a pre-cluster number approximately consistent with the reference cell-type count (0.9), and a higher resolution generating more spatial pre-clusters than the reference annotation (1.2).

For each resolution configuration, we performed the complete ASAOT deconvolution pipeline and collected spot-level cell-type proportion estimates. To comprehensively assess result stability across different pre-clustering hyperparameters, we calculated the spot-wise standard deviation of cell-type fractions, spot-level PCC, and Jensen–Shannon divergence (JSD) among different resolution groups. Global pairwise Pearson similarities of cell-type proportion profiles were further computed to evaluate the overall consistency of deconvolution outputs.

As illustrated in Supplementary Fig. S2, cell-type proportion estimations exhibited minimal variation across the three tested resolutions. High spot-wise correlation and negligible distribution divergence verified that moderate deviations in pre-cluster numbers do not compromise deconvolution accuracy or spatial pattern recovery. These results demonstrate that ASAOT possesses strong robustness against reasonable misspecification of the pre-clustering number. Furthermore, the flexible “resolu_cluster” parameter also enables the pre-cluster number to be selected independently of the scRNA-seq reference annotations, as an alternative to the default procedure in which the pre-cluster number is matched to the number of reference cell types. Users may determine a proper pre-cluster number purely based on ST expression profiles via the elbow method or silhouette coefficient during a reference-free preprocessing step. After identifying a suitable Leiden resolution that yields the desired number of clusters, users can run pre-clustering and feed the resulting spatial-cluster prior into the pipeline. This flexible configuration potentially enables ASAOT to handle datasets in which the cell-type composition of the scRNA-seq reference mismatches that of the spatial sample.

### Benchmark evaluation metrics

To quantify the performance of the ASAOT model on simulated datasets, three metrics are adopted to assess cell-type fraction estimation: the PCC, root mean square error (RMSE), and JSD. Their definitions are provided below:

**PCC:** The PCC is used to measure the degree of linear correlation between predicted and ground-truth cell-type fractions within each spatial spot *i*:


(3) \begin{equation*} \mathrm{PCC}=\frac{\sum_{k=1}^K\left({\hat{F}}_{ik}-{\overline{\hat{F}}}_i\right)\left({F}_{ik}-{\overline{F}}_i\right)}{\sqrt{\sum_{k=1}^K{\left({\hat{F}}_{ik}-{\overline{\hat{F}}}_i\right)}^2}\sqrt{\sum_{k=1}^K{\left({F}_{ik}-{\overline{F}}_i\right)}^2}} \end{equation*}

where ${\hat{F}}_{ik}$ denotes the predicted fraction of cell type *k* at spot *i*, and ${F}_{ik}$ represents the ground-truth fraction of cell type *k* at spot *i*. ${\overline{\hat{F}}}_i=\sum_{k=1}^K\frac{{\hat{F}}_{ik}}{K}$ stands for the average of predicted fractions across all cell types within spot *i*, and ${\overline{F}}_i=\sum_{k=1}^K\frac{F_{ik}}{K}$ denotes the average of ground-truth cell-type fractions at spot *i*. A higher PCC value indicates more accurate estimation.

**RMSE:** The RMSE between predicted and ground-truth cell-type fractions for an individual spatial spot is defined as follows:


(4) \begin{equation*} \mathrm{RMSE}=\sqrt{\frac{1}{K}\sum_{k=1}^K{\left({\hat{F}}_{ik}-{F}_{ik}\right)}^2} \end{equation*}

A lower RMSE value corresponds to higher estimation accuracy.

**JSD:** The JSD is a symmetric extension derived from the KL divergence. It quantifies the dissimilarity between two probability distributions: the predicted cell-type fraction distribution *P* and the ground-truth distribution *Q*. The formal definition of JSD is given below:


(5) \begin{equation*} \mathrm{JSD}\left(P\parallel Q\right)=\frac{1}{2}\mathrm{KL}\left(P\parallel M\right)+\frac{1}{2}\mathrm{KL}\left(Q\parallel M\right) \end{equation*}

where $M=\frac{P+Q}{2}$. A lower JSD value indicates higher estimation accuracy.

**RJP:** a unified ranking-based evaluation metric (RJP) serves as a unified comprehensive metric that integrates the overall ranking information of the three aforementioned evaluation indicators. Specifically, for each independent simulation scenario (e.g. configurations with a fixed cell count or specific tissue type), we first transform RMSE and JSD: let $RMS{E}^{\ast }=1- RMSE$ and $JS{D}^{\ast }=1- JSD$. Together with the original PCC, all three metrics are recast such that larger values correspond to better model performance. Next, under each scenario *s*, we compute the performance of all competing methods on the three transformed metrics and perform ranking separately, yielding three rank values for each method within scenario *s*. The arithmetic mean of these three ranks is taken as the comprehensive rank of the method under scenario *s*. Let $m\in \left\{1,2,\dots, M\right\}$ denote the total number of compared methods. In this work, *M=9*, including ASAOT, Stereoscope [15], RCTD [16], SPOTlight [18], uniPort [22], STRIDE [23], CARD [24], SONAR [25], and SWOT [26]:


(6) \begin{equation*} Ran{k}_m^s=\frac{1}{3}\left(\mathit{\operatorname{rank}}{\left( RMS{E}_m^s\right)}^{\ast }+\mathit{\operatorname{rank}}{\left( JS{D}_m^s\right)}^{\ast }+\mathit{\operatorname{rank}}\left( PC{C}_m^s\right)\right) \end{equation*}

where ${\left( RMS{E}_m^s\right)}^{\ast }$ denotes the $RMS{E}^{\ast }$ value of the *m* method under the *s* scenario, $Ran{k}_s^m$ corresponds to the comprehensive rank derived from the rankings of the three metrics for the *m* method in scenario *s* (Rank 1 represents the best performance). To obtain the overall rank of each method across all experiments, we further average the comprehensive ranks over all scenarios:


(7) \begin{equation*} RJ{P}_m=\frac{1}{S}\sum_{s=1}^S Ran{k}_m^s \end{equation*}

where *S* is the total number of scenarios. In this study, *S=8*, consisting of one Cortex dataset and seven MBAging sub-datasets. A lower value indicates superior overall performance of the method across all scenarios. The RJP metric provides a unified standard independent of metric scales [26], enabling intuitive comparison of the overall robustness of different methods under diverse experimental conditions and mitigating the bias caused by evaluation relying on a single metric. Note that RJP is defined at the simulation-scenario level and is used for overall method ranking. Spot-level pairwise performance comparisons are instead based on spot-level values of the three individual metrics (PCC, 1-RMSE, and 1-JSD).

## Results

In this section, we briefly introduce ASAOT (see Fig. 1). A detailed description of the model can be found in the Methods section.

**Figure 1:**
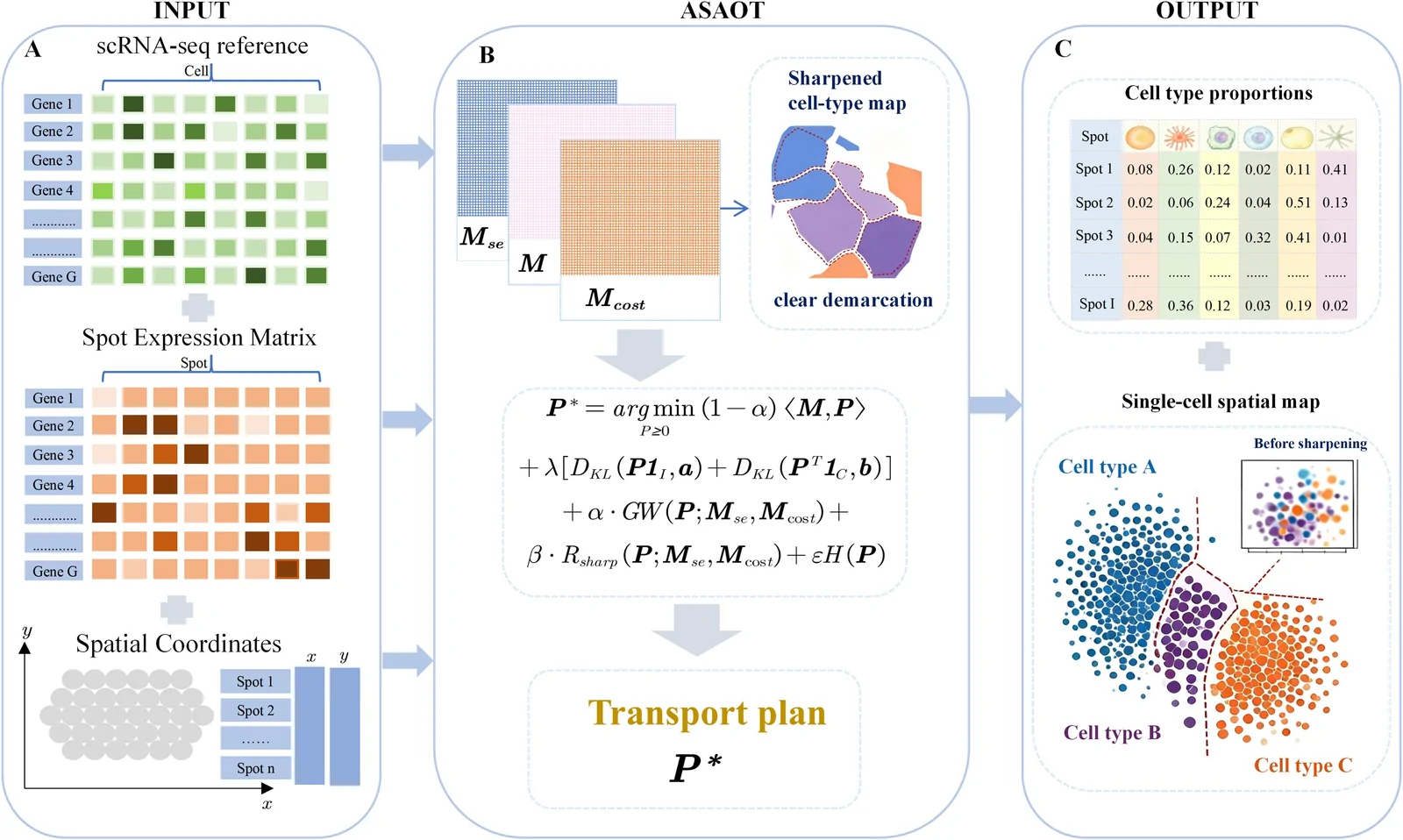
Overview of ASAOT for STs deconvolution; (a) inputs of ASAOT: single-cell transcriptomic data (top), ST gene expression matrix (middle), and spatial coordinate information (bottom); (b) deconvolution model built upon the ASAOT framework; (c) outputs of ASAOT: cell-type fractions for each spatial spot and resolution-adaptive spatial maps of reference single-cell profiles.

The ASAOT model takes three inputs: the gene expression count ${S}_{g c}^{(k)}={\left({x}_{g c}^{(k)}\right)}_{g\epsilon \mathbf{\mathcal{G}},c\in \mathbf{\mathcal{C}}}$ of gene *g* in cell *c* belonging to cell type *k* from single-cell data, the gene expression count ${T}_{gi}$ of gene *g* at spatial spot *i* from STs data, and the spatial coordinate matrix. Spatial deconvolution is implemented via an ASAOT framework, which consists of two core components: the optimal transport module and the cell mapping module.

Within the optimal transport module, three distance matrices are first constructed using single-cell expression profiles, spatial expression profiles, and spot coordinates: the single-cell expression similarity matrix ${\boldsymbol{M}}_{\boldsymbol{se}}$, the cell-to-spot distance matrix $\boldsymbol{M}$, and the enhanced spatial matrix ${\boldsymbol{M}}_{\boldsymbol{cost}}$ integrated with pre-clustering information. The model builds a loss function composed of five terms, whose weights are tuned by hyperparameters. Notably, the differentiated sharpening term carries independent gradients and alleviates boundary blurring induced by conventional global smoothing. The entire optimization problem is solved iteratively by the conditional gradient algorithm combined with the Sinkhorn algorithm, yielding the optimal transport probability matrix ${\boldsymbol{P}}^{\ast}$ that maps single cells to spatial spots. Leveraging the transport probability matrix ${\boldsymbol{P}}^{\ast}$ and cell type labels, the cell mapping module achieves dual analytical goals. Noise reduction is conducted by normalizing transport probabilities to output cell-type fractions at each spatial spot. Meanwhile, gene expression information is assigned to cells within spot neighborhoods according to transport probabilities, enabling reliable spatial mapping of reference single-cell profiles under conventional moderate-resolution STs conditions.

### Benchmarking on simulated datasets

To quantitatively assess the overall performance and spatial-resolution robustness of ASAOT for cell-type fraction deconvolution, benchmark validations are performed on two simulated STs datasets with distinct origins and tissue characteristics. The Simulated_Cortex dataset [26] is constructed from experimental SeqFISH+ data of the mouse somatosensory cortex [34], while the Simulated_MBAging dataset [26] is generated based on MERFISH single-cell STs data of the aging mouse brain [35]. Ground-truth cell-type fractions are available for both datasets, enabling objective and quantitative evaluation of competing algorithms. For Simulated_MBAging, seven sub-datasets with varying numbers of spots are produced by grid-based resampling of the original MERFISH coordinates (spot counts: 290, 479, 749, 989, 2850, 4669, and 9554), to test algorithm stability across different spatial resolutions. These resampled spot densities recapitulate typical spot-density ranges observed across real-world STs platforms: sub-datasets with fewer spots approximate the low-density profiling conditions of 10× Visium, whereas sub-datasets with abundant spots resemble the high-density sampling regime of Slide-seq. Eight widely used spatial deconvolution methods are selected for comparison: Stereoscope [15], RCTD [16], SPOTlight [18], uniPort [22], STRIDE [23], CARD [24], SONAR [25], and SWOT [26]. Three evaluation metrics, namely 1-RMSE, 1-JSD, and PCC, are adopted consistently; higher values indicate superior prediction performance. The comprehensive metric RJP [26], defined as the averaged rank across the three metrics, is utilized to achieve integrated multi-criteria assessment.

The overall ranking of all algorithms is determined by the average RJP values aggregated across all simulated datasets (see Fig. 2a). ASAOT achieves the best average comprehensive rank with a mean score of 2.792, outperforming the baseline method SWOT (3.667), as well as classic deconvolution tools including SONAR (3.562) and STRIDE (4.333). This result indicates that ASAOT obtained the best average comprehensive rank across the selected simulation scenarios. Boxplots illustrating metric distributions separated by dataset reveal comparable performance ranges for RCTD, CARD, SONAR, and ASAOT on the Simulated_Cortex dataset, all exhibiting high median values of PCC, 1-JSD, and 1-RMSE (see Fig. 2b). On the Simulated_MBAging dataset characterized by prominent laminar architecture, ASAOT yields higher median values across all three metrics relative to most competing algorithms, with particularly pronounced advantages in PCC correlation (see Fig. 2c). Pair-wise spot-level comparisons across Simulated_MBAging datasets were computed using Hodges–Lehmann median-difference estimators derived from spot-wise paired values of PCC, 1-RMSE, and 1-JSD (see Supplementary Note S2). Figure 2d presents these descriptive median-difference point estimates. Descriptive median-difference values are fully tabulated in Supplementary Table S2. Positive median-difference values denote better spot-level performance for ASAOT, whereas negative values indicate better spot-level performance for the competing method. Among eight pairwise contrasts, ASAOT showed favorable median-difference values for seven competitors; the comparison versus Stereoscope yielded a negative median difference, meaning Stereoscope exhibited better spot-level performance under these simulation conditions.

**Figure 2:**
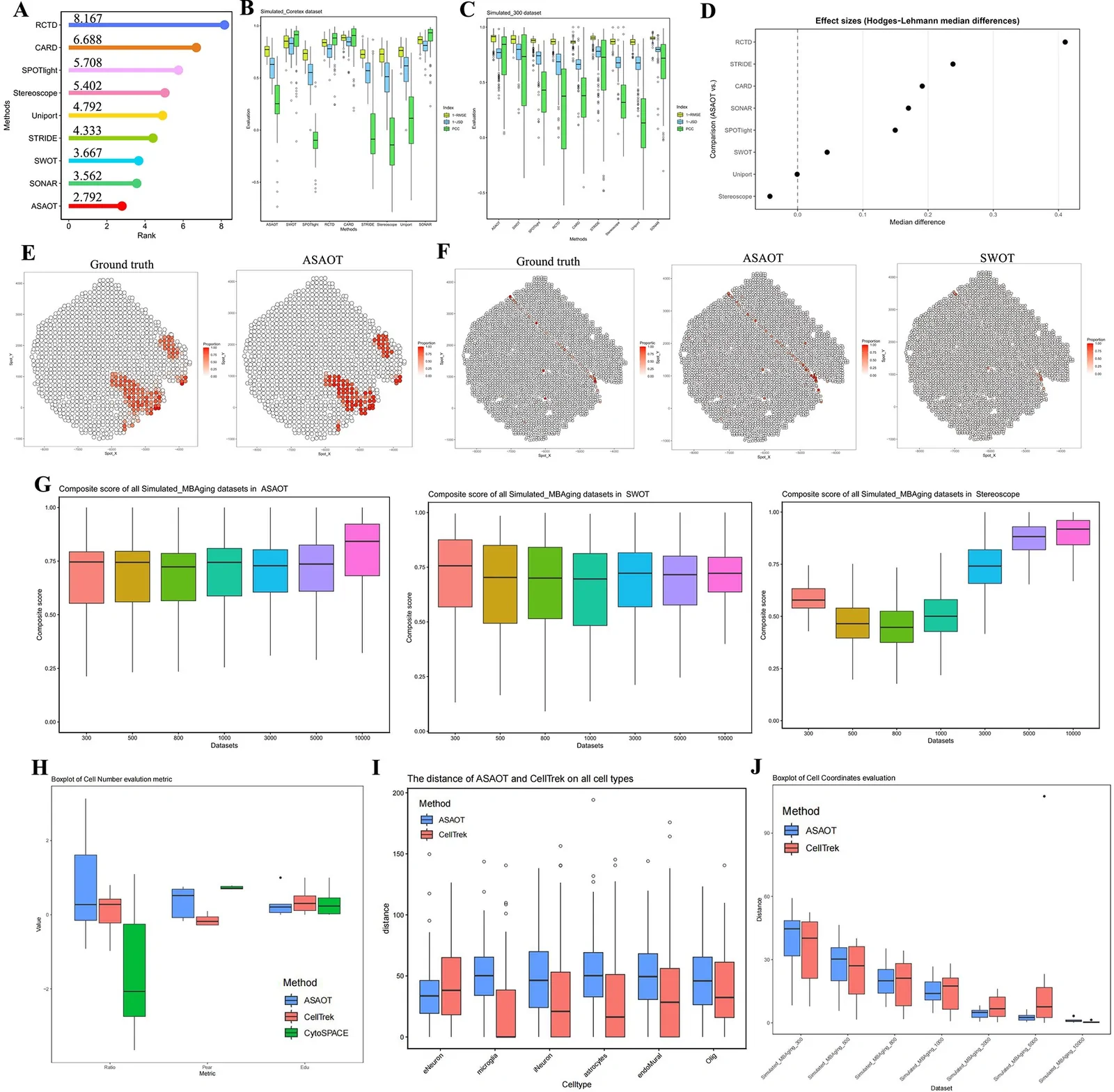
Benchmark comparison between ASAOT and eight competing methods (SONAR, SWOT, STRIDE, uniPort, Stereoscope, SPOTlight, CARD, and RCTD) on two simulated datasets; (a) overall performance ranking of each spatial deconvolution method across all simulated datasets; values denote the average rank of each method over all 24 evaluation conditions (8 simulation scenarios × 3 metrics); lower values indicate superior comprehensive performance; (b) metric evaluation results for all methods on the simulated_Cortex dataset; (c) metric evaluation results for all methods on the Simulated_MBAging_300 dataset; (d) forest plot of effect sizes (Hodges–Lehmann median differences) for ASAOT and eight competing methods; positive median-difference values indicate ASAOT tends to yield higher metric values, and negative values indicate the competing method tends to yield higher metric values; (e) spatial fraction distributions of MSNs from the ground truth (left) and ASAOT predictions (right) on Simulated_MBAging_1000; signal intensity corresponds to cell-type fractions; (f) spatial fraction distributions of VLMC cell type on Simulated_MBAging_3000; from left to right: ground truth, ASAOT predictions, and SWOT predictions; signal intensity indicates the fraction of VLMC cells within each spatial spot; (g) boxplots of comprehensive scores across all spatial spots obtained by ASAOT, SWOT, and Stereoscope on Simulated_MBAging sub-datasets with varying spot counts; the *x*-axis represents simulated datasets containing different numbers of spots; boxes span the first to third quartiles, the middle line inside each box denotes the median, whiskers extend to 1.5 times the interquartile range, and scattered points beyond whiskers correspond to outliers; (h) box-and-whisker plots summarize three quantitative metrics used to evaluate spot-wise total-cell-count predictions, including estimation bias ratio, PCC, and Euclidean distance between predicted and ground-truth cell numbers; (i) box-and-whisker plots display single-cell localization errors, calculated as Euclidean distances between known ground-truth locations and algorithm-inferred spatial coordinates; errors are stratified by cell type using the Simulated_Cortex benchmark dataset; (j) single-cell mapping error distributions across the series of multi-resolution Simulated_MBAging datasets; horizontal-axis labels correspond to individual resampled datasets with distinct spot densities.

To intuitively evaluate the accuracy with which different methods reconstruct the spatial distribution patterns of cells, two representative datasets with distinct resolutions, Simulated_MBAging_1000 and Simulated_MBAging_3000, were selected. We visualized spatial fractions for medium spiny neurons (MSNs), which are highly enriched in laminar layers, and vascular and leptomeningeal cells (VLMCs), which exhibit sparse linear distribution. Spatial reconstruction results from the ground truth, ASAOT, and the baseline SWOT were compared (see Fig. 2e and f). For MSNs forming continuous band-shaped enrichment regions, ASAOT faithfully recapitulates the spatial enrichment domains, magnitude gradients of fractions, and boundary contours seen in the ground truth. By contrast, SWOT suffers from regional signal diffusion and smoothed dilution of local cell proportions. For sparsely distributed VLMCs arranged in linear patterns, conventional SWOT is constrained by global spatial smoothing, resulting in the loss of several high-fraction spots and blurring of cell distribution boundaries. Benefiting from the introduced differentiated sharpening mechanism, ASAOT mitigates signal attenuation caused by global smoothing. It better reproduces the continuous linear layout of VLMCs and achieves superior consistency with scattered high-proportion spots in the ground truth. These visual comparisons demonstrate that ASAOT is adaptable to two typical spatial cellular patterns: continuous laminar enrichment and sparse scattered distribution. Its capability to recover fine spatial details outperforms the conventional optimal transport method SWOT.

We utilized seven Simulated_MBAging sub-datasets with varying spot counts to quantify cell-type distributions across all spatial spots for ASAOT, SWOT, and Stereoscope, and investigate how improved spatial resolution affects deconvolution stability (see Fig. 2g). Boxplots of comprehensive scores for all nine benchmarked methods (ASAOT, Stereoscope, RCTD, SPOTlight, uniPort, STRIDE, CARD, SONAR, and SWOT) are provided in Supplementary Fig. S3. As the number of spatial spots increases and spatial granularity improves, the median comprehensive score of ASAOT rises steadily. ASAOT sustains stable and high-quality deconvolution outcomes under both low resolution (300/500 spots) and ultra-high resolution (10 000 spots), demonstrating exceptional adaptability to varying resolutions. For SWOT, score dispersion increases substantially at higher resolutions; anatomical structures tend to become blurred under high-resolution conditions, leading to insufficient stability when reconstructing fine spatial microstructures. Stereoscope exhibits low median comprehensive scores and severe performance fluctuations on low-spot-count datasets, with improved performance only observed in samples containing abundant spots. Collectively, these findings reveal that ASAOT possesses greater tolerance to shifts in spatial resolution compared with mainstream algorithms, and is compatible with STs profiling acquired at both low and high spot densities. Synthesizing multi-dimensional results including quantitative metric statistics, descriptive pairwise comparisons, cellular spatial visualization, and robustness analysis across resolution gradients, ASAOT achieves comprehensive performance improvements on the two simulated brain tissue datasets. Compared with deconvolution approaches based on conventional regression, Bayesian frameworks, and classical optimal transport, ASAOT yields lower prediction errors and higher correlation for global cell-type fractions. Furthermore, empowered by differentiated sharpening regularization and hierarchical spatial constraints, ASAOT balances global spatial continuity and sharpness of local boundaries, preserving hierarchical and heterogeneous spatial patterns of cells in brain tissue. Accordingly, ASAOT enables high-precision cell fraction decomposition for brain STs data generated at varying sequencing densities.

Beyond cell-type fraction deconvolution, ASAOT also infers spatial coordinates for individual single cells. This single-cell localization pipeline relies on spot-level cell-number initialization. Given the observed resolution-dependent deconvolution performance, we further systematically evaluated the accuracy of both the cell-number estimation and single-cell mapping across the same multi-resolution simulated benchmarks (see Fig. 2h–j). We first compared predicted spot-wise total-cell abundances against ground-truth cell counts across datasets with 300–10 000 spots, using estimation ratio, PCC, and spot-wise Euclidean distance as quantitative metrics (see Fig. 2h and Supplementary Table S1). The cell-number estimator exhibits resolution-dependent performance: cell-count estimates were severely underestimated in the 300-spot condition, remained substantially biased at 500 spots, and exhibited moderate deviation from ground-truth values for the intermediate 800 and 1000 spots datasets. For ultra-large datasets (>3000 spots), the directionality shifted toward overestimation, with substantially elevated estimation bias. In addition, we evaluated downstream single-cell spatial mapping accuracy by calculating Euclidean distances between predicted and ground-truth single-cell coordinates. Cell-type-specific mapping errors on the Simulated_Cortex dataset are presented in Fig. 2i. Cross-resolution mapping performance across all Simulated_MBAging sub-datasets is shown in Fig. 2j. Notably, an opposite resolution trend is observed between upstream cell-count estimation and downstream cell localization. Higher spot density consistently reduces single-cell coordinate error for both ASAOT and CellTrek, indicating that the spatial sampling gain at high resolution outweighs the bias introduced by imperfect cell-number initialization.

We further conducted hyperparameter sensitivity analysis for *β*, the tunable coefficient of the structure-aware sharpening regularization term in ASAOT. We systematically tested a gradient of *β* values (0, 0.001, 0.005, 0.01, 0.05, and 0.1) across all simulated datasets with distinct spatial resolutions and data characteristics. Our results demonstrate that the optimal *β* is adaptively determined by both the intrinsic tissue laminar architecture and the noise level of ST profiles. For high-quality Simulated_Cortex data with extremely clean and inherently sharp layer boundaries, no additional sharpening is required, and disabling the structural regularization *β =0* achieves the optimal deconvolution performance. In contrast, for the relatively noisy layered Simulated_MBAging datasets, a mild positive sharpening strength *β =0.001* effectively compensates for signal diffusion and recovers fine laminar structures, yielding the best overall accuracy across most resolution conditions. Excessively large $\beta \geqslant 0.05$ consistently impairs performance by introducing over-sharpening artifacts and distorting genuine cellular spatial distributions. The comprehensive performance trends are visualized in Supplementary Fig. S4.

### ASAOT resolves spatial patterns of cell types in the mouse olfactory bulb

To map the spatial distribution of diverse cell populations within the MOB and systematically validate the reliability of ASAOT in cellular composition inference, we applied ASAOT to the MOB STs dataset of the MOB [33]. The olfactory bulb exhibits characteristic bilateral symmetry and distinct laminar anatomical architecture. Four principal layers, namely the glomerular layer, mitral cell layer, granule cell layer, and olfactory nerve layer (ONL), are each predominantly populated by signature cell types that shape tissue organization [36]. Guided by H&E-stained histological sections of the MOB, we performed manual anatomical layer annotation (see Fig. 3a) to precisely delineate the boundaries of the four layers.

**Figure 3:**
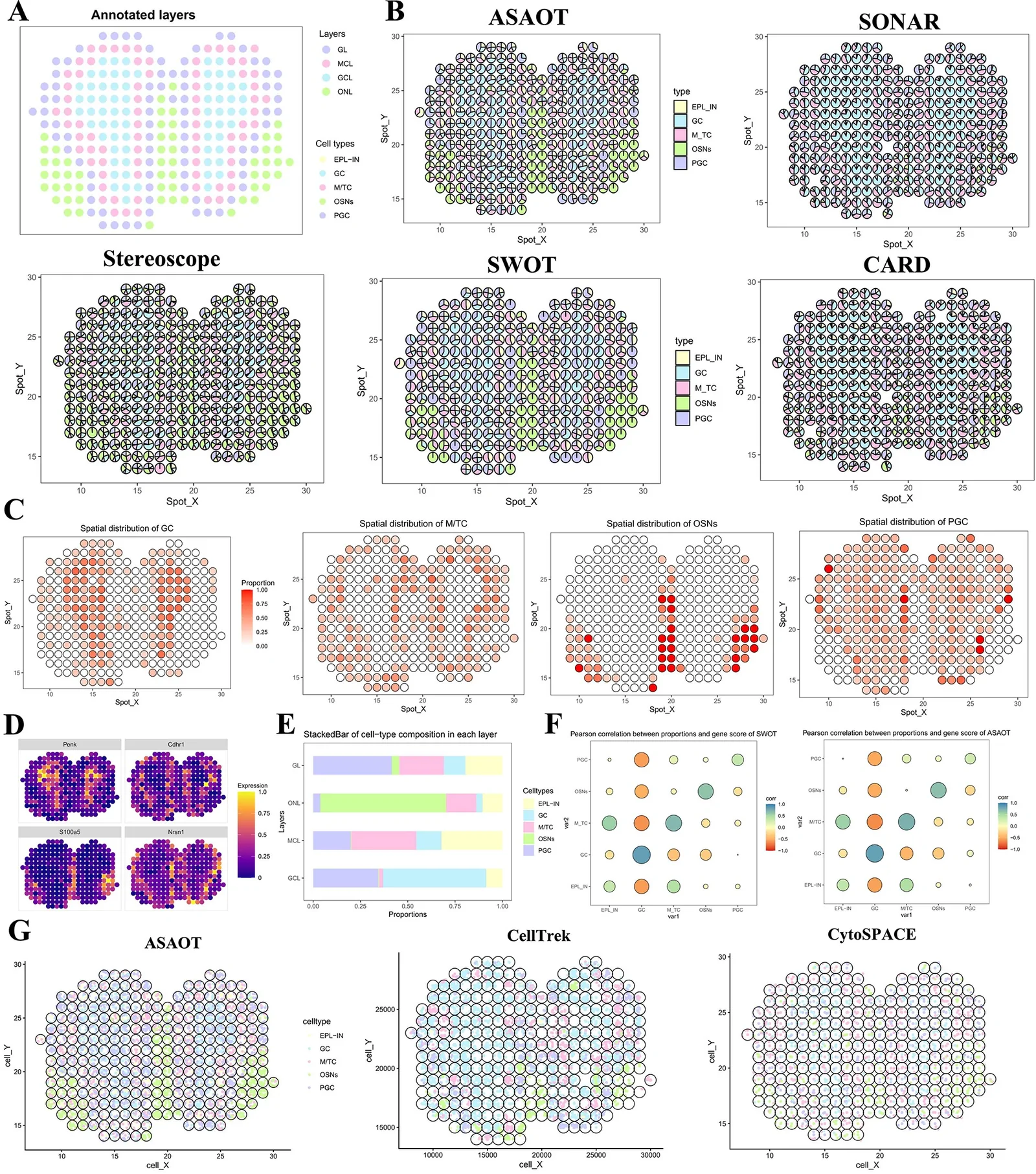
Application of ASAOT on the MOB dataset; (a) spatial annotation of anatomical layers in the olfactory bulb; (b) scatter pie charts of cell-type fractions inferred by five algorithms: ASAOT (top middle), SONAR (top right), Stereoscope (bottom left), SWOT (bottom middle), and CARD (bottom right); (c) spatial distributions of four cell-type fractions (GC, M/TC, OSNs, PGC) estimated by ASAOT; color intensity corresponds to cell fraction within each individual spatial spot; (d) spatial expression patterns of cell-specific marker genes for the four cell types derived from ASAOT; color intensity indicates gene expression levels; (e) cell-type composition across anatomical layers of the MOB predicted by ASAOT; distinct colored segments within each layer represent the fraction of each cell population; (f) Pearson correlations between inferred cell-type fractions and cell signature gene scores obtained from ASAOT (left) and SWOT (right); bubble size denotes the absolute value of the correlation coefficient, and bubble color distinguishes positive and negative correlation; (g) single-cell spatial distributions inferred by ASAOT, CellTrek, and CytoSPACE; this subfigure aims to assess whether spatial-assigned reference single-cell coordinates can reproduce the anatomically defined sharp laminar boundaries of the MOB; Hollow circles denote spots, and colored scattered points represent spatially mapped reference single-cell profiles labeled by cell type.

First, ASAOT effectively identifies the spatial patterns of five cell subtypes, and the enriched regions of cell populations show high concordance with anatomically annotated layers (see Fig. 3b). By contrast, cell-type distributions predicted by Stereoscope and CARD tend to be homogenized across layers, failing to resolve layer-specific signatures. SONAR captures rough laminar trends yet yields blurred boundaries of olfactory sensory neurons (OSNs) in the ONL, and cannot reconstruct fine laminar demarcations. SWOT is generally capable of recovering the four-layer architecture but suffers from mild cross-layer leakage of cellular signals at layer interfaces due to global spatial smoothing. Benefiting from the differentiated sharpening strategy, ASAOT balances global spatial continuity and sharpness of local cellular boundaries. It effectively delineates the laminar enrichment domains of OSNs, granule cells (GCs), mitral/tufted cells (M/TCs), and periglomerular cells (PGCs) while mitigating non-specific cell mixing. ASAOT not only preserves the global continuity of layered tissue architecture but also retains subtle differences in cellular composition at individual spatial spots.

For four key cell populations (GCs, M/TCs, OSNs, and PGCs), we generated spatial heatmaps of cell-type fractions and corresponding spatial expression maps of cell-specific marker genes (see Fig. 3c and d). The laminar distribution trends of cell fractions closely aligned with regions exhibiting high expression of signature genes. For instance, spots with elevated OSN fractions within the ONL simultaneously displayed prominent upregulation of OSN marker genes, verifying the reliability of cell-fraction predictions from ASAOT. We further quantified the average cell-type fraction of all spatial spots within each anatomically annotated layer and constructed stacked bar charts illustrating laminar cellular composition (see Fig. 3e). The layer-wise cell fractions estimated by ASAOT exhibited pronounced differences, reflecting heterogeneous cellular composition across distinct layers. Bubble plots depicting Pearson correlations between cell-type fractions and cell signature gene scores revealed that both ASAOT and SWOT achieved strong positive self-correlations for each cell type, indicating comparable overall quantitative accuracy for cell abundance estimation (see Fig. 3f). Nevertheless, weak positive correlation was observed between GC fractions and PGC signature gene scores in SWOT outputs, suggesting that global smoothing induces signal crosstalk between these two inhibitory neuronal subtypes. In contrast, ASAOT effectively suppresses non-specific cross-correlation across distinct cell subtypes and yields enhanced transcriptional specificity for cell type assignment. Collectively, ASAOT reduces intercellular signal leakage while maintaining accurate cell matching, thereby achieving superior precision in cell-type deconvolution.

At the reference single-cell spatial-mapping level, ASAOT estimates the number of cells residing within each spot based on spot-level expression profiles and further assigns spatial coordinates for all reference single-cell profiles, enabling resolution enhancement from spatial spots down to single cells. We conducted a side-by-side comparison of single-cell localization results generated by ASAOT and two prevalent reference-based single-cell spatial-mapping approaches, CellTrek [12] and CytoSPACE [13] (see Fig. 3g) to assess whether the single-cell spatial arrangement derived from each method is capable of reproducing the anatomically defined sharp laminar boundaries of the MOB. In terms of spatial arrangement, all three methods broadly recapitulate the laminar architecture of the olfactory bulb. Notably, the single-cell layout produced by ASAOT is highly consistent with the laminar enrichment trends derived from deconvolution, exhibiting sharp layer boundaries. In contrast, single-cell distributions produced by CellTrek [12] and CytoSPACE [13] appear more diffuse with weak laminar constraints, leading to limited capability in recovering fine anatomical structures.

Collectively, these analyses demonstrate that the differentiated sharpening mechanism embedded in ASAOT is adaptable to diverse spatial patterns of multiple cell types. Integrating evidence from qualitative visualization, quantitative correlation analysis, layer-wise statistics, and reference single-cell spatial mapping, ASAOT outperforms prevailing deconvolution tools including CARD [24], SONAR [25], and SWOT [26]. It exhibits prominent strengths in predicting laminar cell-type fractions and delineating sharp spatial boundaries, rendering it suitable for accurate profiling of layered brain-tissue spatial cell atlases under moderate-resolution experimental conditions.

### ASAOT resolves fine architecture of the mouse brain

Next, we applied ASAOT to a dataset of the anterior sagittal section of the mouse brain profiled by 10× Visium STs, focusing on high-resolution spatial analysis of the cerebral cortex (see Fig. 4a). A single-cell transcriptomic reference dataset consisting of adult mouse cortical cells generated via Smart-seq2 sequencing was adopted [37]. ASAOT was employed to perform cell-type fraction deconvolution across the entire tissue section, enabling spatial mapping of reference single-cell profiles for diverse neuronal, glial, and vascular cell populations. Global scatter pie plots of cell-type fractions reveal distinct regional segregation of each cell type within the cortex (see Fig. 4b).

**Figure 4:**
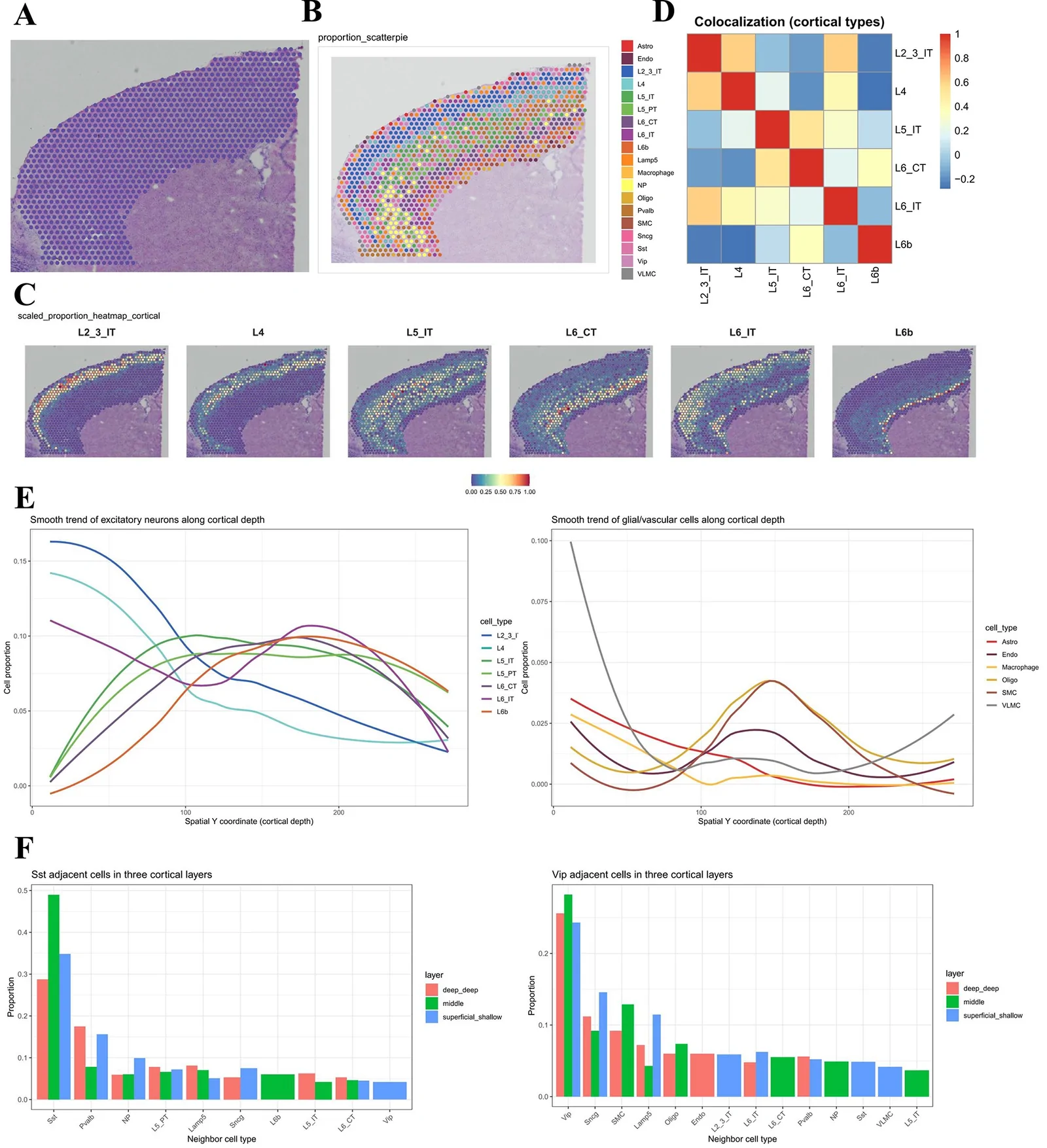
Application of ASAOT on the mouse brain dataset; (a) H&E-stained histological image of the anterior portion of the mouse sagittal brain section; selected spots are marked with circles; (b) spatial scatter pie charts showing cell-type fractions inferred by ASAOT; (c) normalized abundance distributions of cell subtypes inferred by ASAOT; (d) cell-type co-localization patterns characterized by PCCs of cell-type fractions across all spots; (e) smoothed trends of cell abundance along the cortical depth; left: excitatory neurons; right: glial and vascular cell populations; the horizontal axis represents cortical depth (larger Y coordinate corresponds to deeper cortex), and the vertical axis denotes cell-type fraction; (f) bar plots illustrating the proportions of neighboring cells surrounding Sst neurons (left) and Vip neurons (right) in three cortical subregions; the three colors correspond to superficial, middle, and deep cortical layers, and the vertical axis represents the fraction of adjacent cells.

Reference-based spatial mapping was performed with ASAOT using the mouse cortical single-cell reference dataset, and we visualized the normalized abundance distributions of six subtypes of glutamatergic excitatory neurons (see Fig. 4c). L2_3_IT and L4 neurons are predominantly enriched in the superficial cortical layers, L5_IT neurons accumulate in the middle cortex, while L6_CT, L6_IT, and L6b neurons reside within deep cortical regions. These six neuronal subtypes form an ordered laminar architecture along the cortical depth, showing strong consistency with anatomical knowledge of the mouse cortex and published MERFISH spatial atlases [38]. Cell-type co-localization patterns further reveal the spatial organization of cellular populations (see Fig. 4d). Excitatory neurons residing in adjacent cortical layers tend to exhibit significant positive correlation and extensive spatial co-localization, exemplified by superficial L2_3_IT and L4 cells. In contrast, neuron subtypes separated by large depth intervals generally display negative correlation and spatial segregation, such as superficial L2_3_IT/L4 and deep L6b neurons. Serving as an intermediate population, middle-layer L5_IT neurons show neither strong co-occurrence nor repulsion relative to both superficial and deep neuronal subtypes. This weak correlation with both superficial- and deep-layer populations manifests its transitional spatial identity within cortical laminar organization (see Fig. 4d and e). Positioned at the depth interface between upper and lower cortical compartments, L5_IT occupies a cellular transition zone. Our spatial deconvolution results suggest that this population may facilitate cross-layer signal integration within cortical columns, consistent with established laminar organizational principles of the mouse neocortex.

Glial and vascular cell populations also exhibit evident depth-dependent distribution patterns across the cortex. VLMCs are highly enriched in the outermost cortical layer; oligodendrocytes (Oligo) are more abundant in the middle and deep cortex. Astrocytes (Astro), endothelial cells (Endo), vascular smooth muscle cells (SMC), and macrophages generally display higher abundance in the superficial cortex, with reduced levels in the middle cortex. We further performed smoothed fitting of all cell abundances along the cortical depth. The continuous trend curves quantitatively characterize the gradual variation of cell abundance across cortical layers (see Fig. 4e, left). Excitatory neurons exhibit sequential rise and fall along the cortical axis: superficial subtypes L2_3_IT and L4 show steadily decreasing abundance with increasing depth, whereas deep-layer L6 neuronal subtypes gradually increase in abundance. Middle-layer L5_IT and L5_PT form a transitional zone between superficial and deep neuronal populations. Glial and vascular cells also present pronounced depth-dependent distribution signatures (see Fig. 4e, right). VLMCs concentrate heavily in the uppermost cortex, while Astro, Endo, SMC, and macrophages tend to be more abundant in the superficial cortex, with reduced levels in the mid-cortex. These depth gradients of glial and vascular cells align well with known cortical anatomical features. The superficial enrichment of VLMCs is consistent with the native anatomical location of the leptomeninges [38].

To investigate layer-specific spatial adjacency patterns among cortical cell types, we leveraged simulated sub-spot single-cell positions generated by ASAOT. Although the current implementation assigns cells to discrete spatial spots and samples their within-spot coordinates stochastically, we emphasize that our downstream neighborhood analysis is based on probabilistic cell-to-spot assignment rather than exact physical coordinates of individual cells. To quantify the robustness of the inferred adjacency patterns against stochastic coordinate sampling, we repeated the entire coordinate generation and neighborhood quantification procedure 20 times with different random seeds (see Supplementary Fig. S5). For the inhibitory subtypes Sst and Vip, we computed the coefficient of variation (CV) of neighbor proportions for each cell-type pair across the 20 replicates. The median CV values were 0.056 for Sst and 0.077 for Vip, indicating that the estimated adjacency proportions are generally stable across random samplings. However, we note that certain rare cell-type pairs exhibited higher variability (maximum CV up to 0.928 for Sst and 0.867 for Vip), reflecting the inherent uncertainty of neighbor quantification for cell types with low local abundance. This elevated variability does not reflect instability of the ASAOT cell-to-spot assignment, but arises because neighborhoods formed by rare cell populations are inherently sensitive to stochastic within-spot coordinate sampling.

Based on these robustly identified trends, we partitioned the cortex along its depth into three major regions: superficial, middle, and deep layers. We then quantified the compositional fractions of neighboring cells surrounding Sst and Vip neurons (see Fig. 4f). Both Sst and Vip neurons exhibited a pronounced preference for spatial aggregation of cells belonging to the same subtype, with the highest fraction of homotypic neighbors observed in the middle cortical layer. The observed adjacency patterns recapitulate well-established circuit organizational principles in the neocortex: Sst neurons are frequently found in close proximity to parvalbumin (Pvalb) neurons, whereas Vip and Sst neurons tend to spatially segregate, with low probability of direct contact [39, 40]. Furthermore, the neighboring cellular composition of these two inhibitory neurons varied dynamically across cortical depth. In the superficial cortex, Vip neurons were predominantly adjacent to superficial excitatory L2_3_IT neurons. In deep cortical regions, deep-layer L6 excitatory subtypes constituted a larger proportion of cells neighboring both Sst and Vip neurons. Glial and vascular cells occupied a relatively minor fraction in the immediate vicinity of inhibitory neurons, suggesting that neuron–neuron spatial proximity forms the primary component of the local microenvironment surrounding these inhibitory neurons within the cortex. Notably, our core qualitative trends, particularly the major layer-specific enrichment patterns, were consistently observed across all 20 random-seed replicates, confirming that the primary biological conclusions are robust to stochastic coordinate sampling despite sampling-driven uncertainty for a small subset of rare neighbor-cell pairs.

Overall, ASAOT effectively reconstructs the laminar spatial architecture of neurons, glia, and vascular cells across the depth of the mouse cortex. It not only quantifies gradual shifts in cell fractions on a continuous scale, but also enables quantitative analysis of neighboring cell compositions around inhibitory neurons within three partitioned cortical regions using inferred spatial coordinates of reference single-cell profiles. Multi-dimensional evidence from cell-type co-localization, depth-dependent abundance gradients, and layer-resolved cellular adjacency corroborates each other. These findings demonstrate that ASAOT can reliably resolve the intricate spatial microenvironment of brain tissue. Furthermore, quantitative differences in layer-specific adjacency patterns reveal that spatial cellular interactions within cortical circuits are highly position-dependent, forming the structural foundation for functional regionalization of the cerebral cortex.

## Discussion

Driven by advances in STs and single-cell sequencing, *in situ* characterization of tissue cellular architecture has become feasible, yet each has inherent limitations. Single-cell sequencing enables precise cell type annotation but eliminates native spatial positional information, whereas STs preserves tissue topological architecture yet is hampered by spot-level cell mixture ambiguity and insufficient single-cell resolution. Existing deconvolution approaches, including CARD and SONAR, typically employ globally uniform smoothing schemes that fail to accommodate local spatial heterogeneity, rendering their outputs susceptible to sequencing noise and technical dropout artifacts. Consequently, they frequently produce blurred layer boundaries, especially in structurally layered brain tissues.

To overcome these limitations, we proposed an ASAOT model with two core innovations. First, we developed a dual-regularization optimization framework to balance global spatial continuity and local structural edge enhancement. During optimization, global smoothing stabilizes homogeneous intra-layer regions, whereas the sharpening operation preserves genuine spatial heterogeneity at tissue boundaries. The tunable *β* parameter modulates the relative strength of these two terms to prevent over-smoothing or discontinuous spatial mappings. Based on grid-search evaluations across simulated datasets, we empirically recommend *β =0.001* for datasets with prominent spot-to-spot signal diffusion, while *β =0* is suggested for high-quality data with inherently sharp anatomical boundaries. Owing to this dual-design paradigm, ASAOT exhibits strong performance for layered brain tissues characterized by gradual intra-layer transitions and sharp inter-layer boundaries. Second, ASAOT enables resolution-dependent spot-level cell count estimation and subsequent spatial coordinate assignment for reference single-cell profiles, improving spatial resolution under typical moderate-resolution experimental conditions. Systematic benchmarking on simulated datasets with diverse cellular distributions and sequencing depths showed that ASAOT attained the best average comprehensive scenario-level rank among eight mainstream deconvolution methods, achieving strong quantitative accuracy, robustness, and spatial-pattern restoration performance. Further validations on MOB and cerebral cortex datasets verified its practical applicability. ASAOT effectively suppresses inter-cell signal crosstalk, generates cell fraction distributions consistent with signature gene profiles, and reconstructs refined spatial architectures derived from mapped reference single-cell profiles with well-defined laminar boundaries. In the mouse cortex, ASAOT effectively recapitulates depth-dependent layered patterns of excitatory neurons, glial cells, and vascular cells, and quantitatively reveals cell co-localization and exclusion relationships. By leveraging spatial coordinates assigned to reference single-cell profiles, our model further characterizes layer-specific adjacency features of inhibitory neurons, effectively dissecting the fine spatial microenvironment and circuit organization of the cerebral cortex.

Despite the promising performance, this study has several limitations worthy of future improvement. Tailored for hierarchical tissue architectures, ASAOT delivers optimal performance for ordered, layer-structured tissues with distinct boundaries. For disordered heterogeneous tissues (e.g. tumor and inflammatory tissues) without prominent spatial stratification, users may set the sharpening hyperparameter *β =0* to disable the structure-differentiated sharpening term, adapting the model to non-layered spatial patterns. Additionally, ASAOT currently depends on external single-cell references, and its accuracy is affected by the matching degree between reference data and query tissues. Future work will develop reference-free and multi-reference integration modules to enhance model generalizability. Moreover, the present framework only incorporates spatial topological and cell-number constraints without introducing spatial domain priors or developmental temporal information. Integrating spatial domain normalization and batch correction strategies will further improve the stability of cross-batch and low-quality data deconvolution. In summary, ASAOT addresses key technical drawbacks of conventional deconvolution methods, including ambiguous boundaries, signal crosstalk, and insufficient single-cell resolution. With integrated sharpening regularization and reference single-cell spatial-mapping modules, ASAOT achieves accurate and robust spatial mapping of reference single-cell profiles for moderate-resolution STs data, enabling high-quality spatial cell atlas reconstruction in layered brain tissues. Future multi-modal integration and algorithm optimization will extend its application to neural development, pathological mechanism analysis, and spatial biomarker identification, providing a reliable computational tool for high-resolution STs interpretation.

**Key Points**

- Accentuated Structure-differentiated Asymmetric Optimal Transport (ASAOT) is a novel spatial deconvolution method built on asymmetric optimal transport, equipped with a differentiated accentuated sharpening regularization term for resolving complex cell spatial patterns in layered brain tissues.
- Benchmark analyses on both simulated and real datasets of mouse brain demonstrate that ASAOT achieves superior performance in cell-type deconvolution compared with mainstream algorithms across moderate spatial resolutions.
- ASAOT effectively alleviates excessive signal smoothing and boundary distortion, enabling accurate recovery of layered anatomical and stratified cellular distributions in brain tissue under typical experimental resolution conditions.

## Supplementary Material

Supplementary_material_bbag615

## Data Availability

The source-code implementing ASAOT, together with processed simulation outputs and benchmark-related intermediate files for reproducibility, is available at https://github.com/Sunzy-jnu/ASAOT. All datasets used in this study are publicly available. The seqFISH+ data of the mouse somatosensory cortex came from http://linnarssonlab.org/cortex. The single-cell resolution MERFISH data used for generating the Simulated_MBAging dataset and the snRNA-seq data used for reference can be downloaded from https://cellxgene.cziscience.com/collections/31937775-0602-4e52-a799-b6acdd2bac2e. For the MOB dataset, the ST and scRNA-seq data were downloaded from https://www.spatialresearch.org/resources-published-datasets/doi-10-1126science-aaf2403/ and GSE121891, respectively. For the mouse brain dataset, the original scRNA-seq dataset is collected from https://satijalab.org/seurat/articles/spatial_vignette.html, and it can be downloaded from https://www.dropbox.com/s/cuowvm4vrf65pvq/allen_cortex.rds?dl=1. The original STs dataset is collected from https://satijalab.org/seurat/articles/spatial_vignette.html.

## References

[1] Salowka A, Willnow D, Herrera-Oropeza G et al. Spatially organized cellular communities shape functional tissue architecture in the pancreas. *Sci Adv* 2025;11:eadx5791. 10.1126/sciadv.adx5791 PMC12609055 41223257

[2] Cang Z, Nie Q. Inferring spatial and signaling relationships between cells from single cell transcriptomic data. *Nat Commun* 2020;11:2084. 10.1038/s41467-020-15968-5 PMC7190659 32350282

[3] Giorgia B, Sito TG, Chee YS et al. Spatially tunable multiomic sequencing using light-driven combinatorial barcoding of molecules in tissues. *PNAS* 2026;123:e2527896123. 10.1073/pnas.2527896123 PMC13214022 42150070

[4] Ma Y, Dou Z, Zha Y et al. SA2E: spatial-aware auto-encoder for cell type deconvolution of spatial transcriptomics data. *Bioinformatics* 2026;42:btag133. 10.1093/bioinformatics/btag133 PMC13070677 41863296

[5] Ståhl PL, Salmén F, Vickovic S et al. Visualization and analysis of gene expression in tissue sections by spatial transcriptomics. *Science* 2016;353:78–82. 10.1126/science.aaf2403 27365449

[6] Stuart T, Butler A, Hoffman P et al. Comprehensive integration of single-cell data. *Cell* 2019;177:1888–902.e21. 10.1016/j.cell.2019.05.031 PMC6687398 31178118

[7] Chen KH, Boettiger AN, Moffitt JR et al. Spatially resolved, highly multiplexed RNA profiling in single cells. *Science* 2015;348:aaa6090. 10.1126/science.aaa6090 PMC4662681 25858977

[8] Li Z, Li X, Yu X et al. SpaJoint: a transfer learning method for spatial transcriptomics deconvolution. *Brief Bioinform* 2026;27:bbag158. 10.1093/bib/bbag158 PMC13069903 41955028

[9] Longo SK, Guo MG, Ji AL et al. Integrating single-cell and spatial transcriptomics to elucidate intercellular tissue dynamics. *Nat Rev Genet* 2021;22:627–44. 10.1038/s41576-021-00370-8 PMC9888017 34145435

[10] Nitzan M, Karaiskos N, Friedman N et al. Gene expression cartography. *Nature* 2019;576:132–7. 10.1038/s41586-019-1773-3 31748748

[11] Tommaso B, Gabriele S, Lorenzo B et al. Deep learning and alignment of spatially resolved single-cell transcriptomes with tangram. *Nat Methods* 2021;18:1352–62. 10.1038/s41592-021-01264-7 PMC8566243 34711971

[12] Runmin W, Siyuan H, Shanshan B et al. Spatial charting of single-cell transcriptomes in tissues. *Nat Biotechnol* 2022;40:1190–9. 10.1038/s41587-022-01233-1 PMC9673606 35314812

[13] Vahid MR, Brown EL, Steen CB et al. High-resolution alignment of single-cell and spatial transcriptomes with CytoSPACE. *Nat Biotechnol* 2023;41:1543–8. 10.1038/s41587-023-01697-9 PMC10635828 36879008

[14] Gaspard-Boulinc LC, Gortana L, Walter T et al. Cell-type deconvolution methods for spatial transcriptomics. *Nat Rev Genet* 2025;26:828–46. 10.1038/s41576-025-00845-y 40369312

[15] Alma A, Joseph B, Asp M et al. Single-cell and spatial transcriptomics enables probabilistic inference of cell type topography. *Commun Biol* 2020;3:565. 10.1038/s42003-020-01247-y PMC7547664 33037292

[16] Cable DM, Murray E, Zou LS et al. Robust decomposition of cell type mixtures in spatial transcriptomics. *Nat Biotechnol* 2021;40:517–26. 10.1038/s41587-021-00830-w PMC8606190 33603203

[17] Kleshchevnikov V, Shmatko A, Dann E et al. Cell2location maps fine-grained cell types in spatial transcriptomics. *Nat Biotechnol* 2022;40:661–71. 10.1038/s41587-021-01139-4 35027729

[18] ElosuaBayes M, Nieto P, Mereu E et al. SPOTlight: seeded NMF regression to deconvolute spatial transcriptomics spots with single-cell transcriptomes. *Nucleic Acids Res* 2021;49:e50. 10.1093/nar/gkab043 PMC8136778 33544846

[19] Zhou Z, Zhong Y, Zhang Z et al. Spatial transcriptomics deconvolution at single-cell resolution using Redeconve. *Nat Commun* 2023;14:7930. 10.1038/s41467-023-43600-9 PMC10692090 38040768

[20] Meng GW, Luonan C, Xiao FZ. Dual decoding of cell types and gene expression in spatial transcriptomics with PANDA. *Nucleic Acids Res* 2024;52:12173–90. 10.1093/nar/gkae876 PMC11551751 39404057

[21] Romain L, Baoguo L, Hadas K et al. DestVI identifies continuums of cell types in spatial transcriptomics data. *Nat Biotechnol* 2022;40:1360–9. 10.1038/s41587-022-01272-8 PMC9756396 35449415

[22] Cao K, Gong Q, Hong Y et al. A unified computational framework for single-cell data integration with optimal transport. *Nat Commun* 2022;13:7419. 10.1038/s41467-022-35094-8 PMC9715710 36456571

[23] Sun D, Liu ZY, Li TW et al. STRIDE: accurately decomposing and integrating spatial transcriptomics using single-cell RNA sequencing. *Nucleic Acids Res* 2022;50:e42. 10.1093/nar/gkac150 PMC9023289 35253896

[24] Ma Y, Zhou X. Spatially informed cell-type deconvolution for spatial transcriptomics. *Nat Biotechnol* 2022;40:1349–59. 10.1038/s41587-022-01273-7 PMC9464662 35501392

[25] Liu ZY, Wu DF, Zhai WW et al. SONAR enables cell type deconvolution with spatially weighted Poisson-gamma model for spatial transcriptomics. *Nat Commun* 2023;14:4727. 10.1038/s41467-023-40458-9 PMC10406862 37550279

[26] Wang L, Hu Y, Gao L. Inference of cell-type composition and single-cell spatial maps from spatial transcriptomics data with SWOT. *Commun Biol* 2025;8:1611. 10.1038/s42003-025-09001-y PMC12630763 41261170

[27] Luca BA, Steen CB, Matusiak M et al. Atlas of clinically distinct cell states and ecosystems across human solid tumors. *Cell* 2021;184:5482–96.e28. 10.1016/j.cell.2021.09.014 PMC8526411 34597583

[28] Fu L, Wang P, Xu G et al. S3RL: enhancing spatial single-cell transcriptomics with separable representation learning. *Adv Sci* 2026;13:e16178. 10.1002/advs.202516178 PMC13042551 41556263

[29] Mémoli F . Gromov–Wasserstein distances and the metric approach to object matching. *Found Comput Math* 2011;11:417–87. 10.1007/s10208-011-9093-5

[30] Zhao P, Zhu J, Ma Y et al. Modeling zero inflation is not necessary for spatial transcriptomics. *Genome Biol* 2022;23:118. 10.1186/s13059-022-02684-0 PMC9116027 35585605

[31] Wang Y, Song B, Wang SD et al. Sprod for de-noising spatially resolved transcriptomics data based on position and image information. *Nat Methods* 2022;19:950–8. 10.1038/s41592-022-01560-w PMC10229080 35927477

[32] Stickels RR, Murray E, Kumar P et al. Highly sensitive spatial transcriptomics at near-cellular resolution with slide-seqV2. *Nat Biotechnol* 2021;39:313–9. 10.1038/s41587-020-0739-1 PMC8606189 33288904

[33] Tepe B, Hill MC, Pekarek BT et al. Single-cell RNA-seq of mouse olfactory bulb reveals cellular heterogeneity and activity-dependent molecular census of adult-born neurons. *Cell* 2018;25:2689–703.e3. 10.1016/j.celrep.2018.11.034 PMC6342206 30517858

[34] Eng CHL, Lawson M, Zhu Q et al. Transcriptome-scale super-resolved imaging in tissues by RNA seqFISH+. *Nature* 2019;568:235–9. 10.1038/s41586-019-1049-y PMC6544023 30911168

[35] Allen WE, Blosser TR, Sullivan ZA et al. Molecular and spatial signatures of mouse brain aging at single-cell resolution. *Cell* 2023;186:194–208.e18. 10.1016/j.cell.2022.12.010 PMC10024607 36580914

[36] Wang LY, Hu YX, Gao L. Adjustment of scRNA-seq data to improve cell-type decomposition of spatial transcriptomics. *Brief Bioinform* 2024;25:bbae063. 10.1093/bib/bbae063 PMC10939420 38426323

[37] Tasic B, Menon V, Nguyen TN et al. Adult mouse cortical cell taxonomy revealed by single cell transcriptomics. *Nat Neurosci* 2016;19:335–46. 10.1038/nn.4216 PMC4985242 26727548

[38] Zhang M, Eichhorn SW, Zingg B et al. Spatially resolved cell atlas of the mouse primary motor cortex by MERFISH. *Nature* 2021;598:137–43. 10.1038/S41586-021-03705-X PMC8494645 34616063

[39] Pfeffer CK, Xue M, He M et al. Inhibition of inhibition in visual cortex: the logic of connections between molecularly distinct interneurons. *Nat Neurosci* 2013;16:1068–76. 10.1038/nn.3446 PMC3729586 23817549

[40] Jiang XL, Shen S, Cadwell CR et al. Principles of connectivity among morphologically defined cell types in adult neocortex. *Science* 2015;350:aac9462. 10.1126/science.aac9462 PMC4809866 26612957

