# Supplementary material for: ASAOT: an accentuated structure-differentiated asymmetric optimal transport model for cell type and single-cell spatial mapping
Source: Brief Bioinform. 2026 Nov 13;27(6):bbag615. doi: 10.1093/bib/bbag615 (PMC13766683; doi:10.1093/bib/bbag615)
Supplement: Supplementary file 1 [file supplementary_material_bbag615.docx]

**Supplementary Material for ASAOT: An Accentuated Structure-differentiated Asymmetric Optimal Transport Model for Cell Type and Single-Cell Spatial Mapping**

Ziyan Sun^1^, Yuan Zhang^1^, Zhixin Shi^1^, Mengdi Nan^1^, Jie Gao^1∗^

1 School of Mathematics and Data Science, Jiangnan University, Wuxi 214122, Jiangsu Province, P. R. China

*Corresponding author: Jie Gao. E-mail address: gaojie@jiangnan.edu.cn

**Supplementary Note S1. Supplementary structured enhanced spatial distance matrix construction**

To fully excavate the intrinsic structural characteristics of spatial transcriptomic data during optimal transport optimization, a multi-dimensional structural constraint strategy is proposed in this study. The Euclidean distance between spatial spots only reflects physical positional differences on tissue slices. Nevertheless, biological tissues commonly exhibit high spatial heterogeneity and layered distribution patterns. Simply relying on physical coordinates to construct spot correlations tends to ignore inherent transcriptional differences, inevitably causing structural distortion and boundary blurring in cell-type estimation. To overcome this limitation, we integrate spatial location and gene expression information to establish a novel structured spatial distance matrix for accurate quantitative modeling of spot-to-spot relationships.

Prior to distance calculation, unsupervised pre-clustering is performed on all spatial spots using the Leiden algorithm. By default, the clustering resolution is dynamically adjusted to ensure that the number of clustered subgroups matches the predefined cell-type quantity, and each spot is assigned an exclusive cluster label. As an optional alternative, users may set the Leiden resolution independently of reference cell-type counts: the pre-cluster number can be selected purely from spatial transcriptomics expression profiles using the elbow method or silhouette coefficient in a reference-free preprocessing step, consistent with the robustness analysis described in the main manuscript. According to label consistency, all spots are categorized as homogeneous or heterogeneous samples relative to the target spot.

To achieve adaptive distance-weight decay with feature differences, two complementary distance metrics, spatial coordinate distance and transcriptional expression distance, are simultaneously adopted. The bi-square kernel function is utilized to compute adaptive basic spatial weights:

 (1)

The hyperparameter denotes the spatial bandwidth that defines the range of local spatial interaction. Specifically, represents the basic weight within the bandwidth, while corresponds to the weight beyond the bandwidth. Different from conventional spatial truncation strategies that directly zero out distant weights, our method retains valid weights for out-of-bandwidth samples based on expression similarity, preventing the loss of long-range homogeneous structural information.

Based on spatial distance threshold and cluster label consistency, pairwise spot relationships are divided into four distinct modes to construct the refined adaptive spatial weight matrix:

 (2)

The hyperparameter flexibly adjusts the weight decay rate for heterogeneous spots within local neighborhoods. This design guarantees valid structural constraints for most spot pairs, suppresses structural noise caused by unstable neighborhood scales and clustering bias, and effectively preserves long-range homogeneous spatial correlations.

On the basis of adaptive spatial weighting, the structured enhanced distance matrix is finally established for optimal transport calculation:

 (3)

where controls the distance scaling degree for homogeneous spots outside the bandwidth. This composite distance integrates dual features of spatial geometry and transcriptional profiles, enabling dynamic constraint differentiation between spatially adjacent heterogeneous pairs and distantly homogeneous pairs. Instead of adopting infinite distance for out-of-bandwidth heterogeneous pairs, continuous random values within are assigned to ensure smooth distance distribution.

The proposed multi-modal structural distance precisely characterizes the genuine biological correlations among spatial spots in heterogeneous tissues, providing robust and refined spatial structural priors for the optimal asymmetric transport model and substantially improving the accuracy of cell-type decomposition and spatial reconstruction.

**Supplementary Note S2. Statistical analysis for Simulated_MBAging datasets.**

(1) Rationale for blocked analysis

The Simulated_MBAging benchmark consists of seven resolution variants (300, 500, 800, 1000, 3000, 5000, and 10,000 spots), all derived from the same underlying MERFISH data via grid-based resampling of the original spatial coordinates. These seven variants are not biologically independent replicates and exhibit strong statistical dependence; treating them as independent would introduce pseudo-replication and inflate the statistical significance of the comparisons. To appropriately account for this nested data structure, we adopted a blocked analysis approach, treating each resolution variant as a separate block. Because of intrinsic dependence among blocks, formal hypothesis testing aggregating across these seven blocks cannot reliably control the type-I error rate; therefore, our primary interpretation relies on descriptive effect-size estimates rather than aggregated significance testing.

(2) Spot-wise paired Wilcoxon signed-rank tests

Within each resolution variant, we performed spot-wise paired Wilcoxon signed-rank tests comparing ASAOT with each competing method. The pairing was conducted at the level of individual spatial spots: for each spot, we obtained spot-level values of the three base evaluation metrics (PCC, 1-RMSE, and 1-JSD) from ASAOT and from the competitor method, and computed the paired difference. This design ensures that spatial heterogeneity (e.g., differences in local cell density, tissue architecture, or sequencing depth across spots) is controlled for, as each spot serves as its own control. Only the relative performance difference between two methods on the same spot is evaluated. For each method pair (ASAOT vs competitor) and each resolution variant, the paired test yielded a p-value, representing the statistical significance of the observed performance difference within that specific resolution.

(3) Fisher's combined probability test

To combine evidence across the seven non-independent resolutions while avoiding pseudo-replication, we applied Fisher's combined probability test. For each method pair, the test statistic was computed as:

 (4)

where are the p-values obtained from the seven individual resolution-specific paired tests. Under the null hypothesis of no overall difference between the two methods across all resolutions, this statistic follows a chi-square distribution with degrees of freedom. We do not interpret this aggregated statistic as formal hypothesis‑testing evidence; effect‑size estimates are prioritized for assessing practical performance differences.

(4) Effect sizes and confidence intervals

Beyond hypothesis‑testing procedures which are invalid given statistical dependence among Simulated_MBAging resolution variants, we quantified performance differences using descriptive effect‑size estimates. Only the Hodges‑Lehmann median‑difference point estimates are reported for descriptive purposes. Pooled confidence intervals are omitted because the seven resolution variants are not statistically independent, and thus do not support valid population‑level statistical inference. This estimator is robust to outliers and provides a meaningful measure of the central tendency of the performance difference. Interpretation of effect sizes: Positive median differences indicate that ASAOT yields higher metric values relative to the competitor; negative median differences indicate that the competitor yields higher metric values relative to ASAOT.

(5) Implementation

All statistical analyses were performed using the R software environment (version 4.3). The paired Wilcoxon tests were conducted using the *wilcox.test()* function with the *paired = TRUE* argument. Although Fisher’s combined‑probability test, Benjamini‑Hochberg correction and Hodges‑Lehmann confidence‑interval calculations were also implemented in code, these outputs are not presented, as statistical dependence among resolution variants prevents valid population‑level inference.

(6) Results summary

Descriptive Hodges‑Lehmann median‑difference point estimates are tabulated in **Supplementary Table S2** and visualized in the forest‑plot inset of **Figure 2D**. All biological interpretations are based solely on these descriptive point estimates. Formal hypothesis‑testing calculations were computed but are not reported, as the non‑independent nature of Simulated_MBAging resolution variants precludes valid statistical inference.

**Supplementary Figure S1. Relationship between cell number and zero proportion**

**per spot.**

Scatter plot showing the empirical negative association between ground‑truth cell numbers and the fraction of zero‑read counts per spot across multiple simulated benchmark datasets (Simulated_Cortex, Simulated_MBAging_300/500/800/1000/3000/5000/10000). Each dot represents an individual spatial spot. The black curve denotes our fitted log‑linear model, whose hyperparameters (slope = −10, intercept = −1) were empirically tuned to recapitulate this broad negative trend between cellular density and zero‑gene proportion. This trend serves as the biological motivation for our initial spot‑wise cell‑number estimation.

**Supplementary Figure S2. Robustness of ASAOT deconvolution results under different pre‑clustering resolutions on the mouse olfactory bulb (MOB) dataset.**

Standard deviation of estimated cell‑type proportions across three Leiden resolution settings (`resolu_cluster` = 0.5, 0.9, 1.2) for five annotated cell types (EPL‑IN, GC, M/T‑C, OSNs, PGC). The Kruskal‑Wallis test was performed to compare the variability of proportion estimates among cell types.

Spot‑wise consistency metrics across the three pre‑clustering resolutions. Spot_JSD quantifies the divergence of cell‑type proportion estimates, while Spot_mean_PCC evaluates the Pearson correlation of deconvolution results at individual spatial spots.

Pair‑wise Pearson similarity heatmap comparing global cell‑type proportion profiles obtained under three different Leiden resolutions (Leiden_Less = 0.5, Leiden_Equal = 0.9, Leiden_Greater = 1.2). High similarity values indicate stable deconvolution outputs with moderate misspecification of the pre‑cluster number.

**Supplementary Figure S3. Boxplots of comprehensive scores across all spatial spots obtained by ASAOT, Stereoscope, RCTD, SPOTlight, Uniport, STRIDE, CARD, SONAR and SWOT on Simulated_MBAging datasets with varying spot counts.**

The x-axis represents simulated datasets containing different numbers of spots. Boxes span the first to third quartiles, the middle line inside each box denotes the median, whiskers extend to 1.5 times the interquartile range, and scattered points beyond whiskers correspond to outliers.

**Supplementary Figure S4. Parameter sensitivity analysis of the ASAOT hyperparameter** **across simulated spatial transcriptomics datasets.**

We systematically evaluated deconvolution performance across six candidate values of the sharpening hyperparameter(0, 0.001, 0.005, 0.01, 0.05, 0.1) on eight independent simulated datasets. Performance was quantified using the average comprehensive rank derived from three core metrics: PCC, 1-RMSE and 1-JSD. The y-axis shows average rank, with lower values indicating superior deconvolution accuracy. Separate panels display trends for Sim_Cortex and seven Simulated_MBAging sub-datasets containing 300, 500, 800, 1000, 3000, 5000 and 1000 spatial spots, respectively. The red line tracks how average performance rank shifts as increases.

**Supplementary Figure S5. Stability analysis of neighbourhood‑proportion estimates across 20 repeated within‑spot coordinate‑sampling runs with distinct random seeds.**

Distributions of neighbourhood cell‑type proportions for Sst‑positive and Vip‑positive inhibitory neurons across twenty replicate samplings. Each dot represents the neighbourhood proportion value obtained from one independent random‑seed run. Points are grouped according to the cortical layer of the reference inhibitory cell.

Coefficients of variation (CV) calculated for each cell‑type‑pair neighbourhood proportion across 20 replicates. The red dashed horizontal line marks a CV threshold of 0.1. Most pairwise neighbourhood proportions show low variation (CV < 0.1), indicating high reproducibility. A small subset of rare cell‑type pairs exhibits elevated CV values, reflecting sampling‑driven uncertainty for low‑abundance cellular populations.

**Supplementary Table S1. Performance comparison of spot‑level cell‑number estimation among ASAOT, CellTrek and CytoSPACE across seven multi‑resolution Simulated_MBAging datasets.**

Ratio: estimation bias ratio, calculated as (predicted cell count / ground‑truth cell count) − 1; Pearson: Pearson correlation coefficient between predicted and ground‑truth spot‑level cell numbers; Euclidean: Euclidean distance between predicted and ground‑truth cell‑count values for each spot. Dataset labels indicate the total number of spatial spots in each simulated sample.

| Dataset (spot count) | **ASAOT** | | | **CellTrek** | | | **CytoSPACE** | | |
| --- | --- | --- | --- | --- | --- | --- | --- | --- | --- |
|  | Ratio | Pearson | Euclidean | Ratio | Pearson | Euclidean | Ratio | Pearson | Euclidean |
| **Simulated_MBAging_300** | -0.918 | 0.518 | 42.941 | 0.143 | 0.098 | 28.897 | -3.653 | 0.688 | 67.645 |
| **Simulated_MBAging_500** | -0.382 | 0.628 | 15.223 | 0.279 | -0.180 | 19.000 | -3.043 | 0.695 | 39.098 |
| **Simulated_MBAging_800** | 0.078 | 0.756 | 4.876 | 0.383 | -0.280 | 11.401 | -2.445 | 0.697 | 23.346 |
| **Simulated_MBAging_1000** | 0.273 | 0.748 | 4.298 | 0.459 | -0.282 | 9.496 | -2.068 | 0.714 | 16.621 |
| **Simulated_MBAging_3000** | 1.188 | -0.147 | 8.224 | 0.801 | -0.272 | 5.649 | -0.611 | 0.768 | 2.939 |
| **Simulated_MBAging_5000** | 2.034 | -0.171 | 12.345 | -0.974 | -0.118 | 2.662 | 0.104 | 0.792 | 0.964 |
| **Simulated_MBAging_10000** | 3.132 | -0.009 | 15.349 | -0.587 | 0.004 | 1.059 | 1.096 | 0.759 | 2.304 |

**Supplementary Table S2. Pairwise statistical comparisons between ASAOT and eight competing methods across all Simulated_MBAging datasets.**

Median Difference: Hodges–Lehmann estimator of the paired median difference (ASAOT score minus competitor score). Positive values indicate that ASAOT yields higher metric values relative to the competitor; negative values indicate that the competitor yields higher metric values relative to ASAOT.

| **Method Comparison** | **Median Difference** |
| --- | --- |
| ASAOT vs SWOT | 0.0579 |
| ASAOT vs SPOTlight | 0.1733 |
| ASAOT vs RCTD | 0.4300 |
| ASAOT vs CARD | 0.2102 |
| ASAOT vs STRIDE | 0.2712 |
| ASAOT vs Stereoscope | -0.0433 |
| ASAOT vs Uniport | 0.0029 |
| ASAOT vs SONAR | 0.1885 |
